# Influence of Prenatal Exercise on Apgar Score and Health‐Related Physical Fitness in Childhood: A Systematic Review and Meta‐Analysis

**DOI:** 10.1155/jp/6702095

**Published:** 2026-09-03

**Authors:** Sandra Sánchez-Parente, Ángela Rodríguez-Perea, Sandra Solaguren-Beaskoa, Laura Baena-García, Virginia A. Aparicio, Linda E. May, José Castro-Piñero

**Affiliations:** ^1^ GALENO Research Group, Department of Physical Education, Faculty of Education Sciences, University of Cadiz, Puerto Real, Spain, uca.es; ^2^ Biomedical Research and Innovation Institute of Cádiz (INIBICA), Cádiz, Spain; ^3^ Faculty of Physical Activity and Sports Sciences, Department of Physical Sport and Education, University of Leon, Leon, Spain; ^4^ Strength and Conditioning Laboratory, CTS-642 Research Group, Department of Physical Education and Sports, Faculty of Sports Science, University of Granada, Granada, Spain, ugr.es; ^5^ Department of Physiology, Institute of Nutrition and Food Technology, Biomedical Research Centre, University of Granada, Granada, Spain, ugr.es; ^6^ Department of Nursing, Faculty of Health Sciences, University of Granada, Granada, Spain, ugr.es; ^7^ Sport and Health University Research Institute (iMUDS), University of Granada, Granada, Spain, ugr.es; ^8^ Biosanitary Research Institute of Granada (ibs.GRANADA), Granada, Spain; ^9^ Department of Kinesiology, College of Health and Human Performance, East Carolina University (ECU), Greenville, North Carolina, USA; ^10^ Department of Obstetrics and Gynecology, Brody School of Medicine, East Carolina University (ECU), Greenville, North Carolina, USA

**Keywords:** child, exercise, physical fitness, pregnancy

## Abstract

**Introduction:**

Adequate health‐related physical fitness (HRPF) in early years is essential for an optimal future quality of life. Consequently, exploring whether prenatal exercise can improve it in the descendants is of undoubted public health interest.

**Purpose:**

The aim of this study is to analyze the influence of prenatal exercise on Apgar score and childhood HRPF and to identify possible differences based on maternal age and pre‐pregnancy body mass index.

**Materials and Methods:**

The search was conducted on PubMed and Web of Science databases. Through PRISMA flow diagram, studies that met the inclusion criteria were selected. Risk of bias and quality of evidence were conducted using Cochrane and GRADE, respectively. We performed a meta‐analysis using Review Manager, JASP, and Jamovi. Heterogeneity was assessed by the Cochran‐Q statistic, and effect size was conducted with a random‐effects model. Sensitivity analysis was performed through casewise diagnostic analysis, and publication bias was evaluated through visual inspection of funnel plots and Egger′s regression test.

**Results:**

Sixty‐nine studies were included in the systematic review and 52 in the meta‐analysis. Apgar score at 1 min was higher in neonates whose mothers performed overall, resistance, and concurrent exercise during gestation (*p* = 0.001; *p* = 0.04; *p* = 0.03, respectively). Overall exercise during pregnancy was associated with better Apgar score at 5 min (*p* = 0.04). Overall and aerobic exercise during pregnancy reduced body birth weight within normal ranges (*p* = 0.02; *p* = 0.009). No significant differences between groups were found in other outcomes.

**Conclusion:**

Exercise during pregnancy improves Apgar score at 1 and 5 min and reduces body birth weight within normal values. Further follow‐up studies assessing the influence of exercise interventions on children′s HRPF are needed.

## 1. Introduction

Apgar score is a tool used worldwide to assess the overall health status of neonates at 1 and 5 min after birth; Apgar score includes the evaluation of muscle tone, heart rate, reflex irritability, skin tone, and respiratory rate [1]. Low Apgar scores (less than 7) are associated with an increased risk of neonatal death and with neurologic disability, including cerebral palsy, epilepsy, and cognitive impairment in childhood and early adulthood [2].

Health‐related physical fitness (HRPF) components, such as cardiorespiratory fitness, muscular fitness, motor fitness, flexibility, and body composition [3], are powerful markers of health [4, 5]. Higher levels of HRPF are related to better self‐rated health [6], bone health [7], psychological health [8], cardiovascular or metabolic profile [5], lower levels of total and abdominal adiposity [9, 10] and higher academic performance [11] in children. In addition, the scientific evidence supports that improvements in HRPF in early years are essential for the development of quality of life in adolescence and adulthood [4, 5].

In the absence of contraindications, women are encouraged to be physically active during pregnancy and postpartum to improve maternal and fetal health outcomes. Exercise during pregnancy has been shown to contribute positively to a better metabolic intrauterine environment [12], reducing the risk of macrosomia (body birth weight > 4000 g), excessive gestational weight gain, and preterm birth [13]. Moreover, it seems that there is a positive influence of exercise at the recommended levels during pregnancy on offspring outcomes, according to the American College of Obstetricians and Gynecologists (ACOG) [14].

Knowing whether exercise during pregnancy may improve all or some components of offspring Apgar score and HRPF would be of great importance from a public health point of view. However, there is a lack of evidence assessing the effect of exercise during pregnancy on the cardiorespiratory fitness, muscular fitness, and flexibility of children. Clapp [15] reported higher scores in neuromotor skills of children exposed to prenatal aerobic exercise (AE) compared with children of women in the control group. However, not much is known about the influence of prenatal exercise on children′s motor fitness.

Although there are some systematic reviews, which evaluated the influence of prenatal exercise interventions on Apgar score and body composition at birth, they have some limitations. To begin, none of the previous systematic reviews have distinguished between AE, resistance exercise (RE) or their combination, known as concurrent exercise (CE). Secondly, regarding body composition, only neonates′ body birth weight was assessed. Thirdly, some reviews only were focused on overweight or obese women with low levels of physical activity and with gestational diabetes mellitus. Lastly, some reviews only assessed the combined effect of diet plus exercise during pregnancy, rather than exercise with and without diet.

Therefore, the primary purpose of the present systematic review and meta‐analysis was to collect and analyze the combined or independent effect of prenatal overall, AE, RE, and CE interventions, with or without diet, on all domains of offspring Apgar score and HRPF up to 5 years of age. A secondary purpose of the analysis was to identify potential differences between Apgar score and HRPF depending on maternal age and pre‐pregnancy body mass index (BMI).

## 2. Materials and Methods

The review was registered in PROSPERO (Registration Number: CRD42022318735) and the applied methodology followed the guidelines drawn in the Preferred Reporting Items for Systematic Reviews and Meta‐Analysis (PRISMA) statement [16].

### 2.1. Literature Search

The search was performed in PubMed and Web of Science (WOS) electronic databases from inception until December 2024. We screened intervention studies that analyzed the influence of exercise in healthy women during pregnancy on the Apgar score and HRPF of offspring up to 5 years. The search was restricted to articles published with humans in English or Spanish.

Therefore, the search descriptors were grouped according to pregnancy, training, exercise, and physical fitness/body composition. The search syntax was adapted to the indexing terms of each database (Table S1).

The PubMed search used a combination of keywords and MeSH terms, whereas WOS used keywords, selecting “All databases” as a database (Table S2). Regarding the search field, the “Topic” filter was used.

### 2.2. Eligibility Criteria

After determining the search strategy, inclusion and exclusion criteria were applied to selected studies for review.

The inclusion criteria were as follows: (1) Experimental studies that include a prenatal intervention program of exercise with or without diet, published in English or Spanish. (2) Full‐text studies that analyzed the effect of a prenatal intervention program of exercise with or without diet during pregnancy on Apgar score and/or body composition, cardiorespiratory fitness, muscular fitness, motor fitness, flexibility in offspring up to 5 years of age.

The exclusion criteria were as follows: (1) Studies whose main sample had high‐risk pregnancies according to Meah et al. [17]; severe respiratory diseases (chronic obstructive pulmonary disease, restrictive lung disease, and cystic fibrosis), severe acquired or congenital heart disease with exercise intolerance, uncontrolled or severe arrhythmia, placental abruption, vasa previa, uncontrolled Type 1 diabetes, intrauterine growth restriction, active preterm labor, and severe pre‐eclampsia and cervical insufficiency. (2) Letters to the editor, pilot studies, abstracts, and case‐control studies.

### 2.3. Data Extraction

All stages of the process were performed in accordance with the PRISMA statement [16]. Two authors (S.S‐P. and S.S‐B.) independently assessed the titles and abstracts of the articles retrieved by the search strategy for eligibility. Then, the full texts of the selected studies were independently screened to determine whether to include based on the inclusion criteria. When no consensus was reached between both researchers, a third researcher (J.C‐P.) made the final decision. Reasons for the exclusion of identified studies were recorded.

### 2.4. Criteria for Risk of Bias Assessment

The risk of bias for the studies included in the systematic review was assessed using The Cochrane Collaboration′s revised tool [18], which was made for each eligible study by two researchers (S.S‐P. and S.S‐B.), independently. Judgments were classified as “low risk of bias,” “some concerns,” and “high risk of bias,” and discrepancies were resolved in a consensus meeting. Interrater agreement for the risk of bias between researchers was calculated by the percentage agreement (94%; Kappa = 0.943). A further reviewer (J.C‐P.) to adjudicate unresolved disagreements was done as needed.

### 2.5. Levels of Evidence

The quality of the studies included in the meta‐analysis was evaluated based on the criteria of the grading of recommendations, assessment, development, and evaluation (GRADE) system: risk of bias, inconsistency of the results, indirectness and imprecision of the evidence [19]. GRADE offers four levels of evidence: high, moderate, low, and very low. In the meta‐analysis, we wanted to assess all outcomes included in our objective (Apgar score, body composition, cardiorespiratory fitness, muscular fitness, motor fitness, and flexibility); however, due to insufficient data, we could only analyze Apgar score and some components of neonatal body composition at birth. For each variable (Apgar score at 1 and 5 min, body birth weight and length and head circumference at birth), we followed the rating structure proposed by the GRADE guidelines [20–22].

Risk of bias assessment from the body of the evidence for each outcome was categorized as “not serious” (if the majority of the studies included had low risk of bias and some concerns), “serious” (if the majority of the evidence had some concerns and high risk of bias) and “very serious” (if the majority of the studies presented a high risk of bias). Serious risk of bias decreases the quality evidence by one level, and very serious risk of bias decreases the quality of evidence by two levels [20]. Since we included experimental studies, indirectness and imprecision of the evidence was “not serious” in all domains assessed [21]. To mitigate the impact of this risk on our final estimates, any potential bias stemming from these studies was further scrutinized through the sensitivity and casewise diagnostic analyses.

*I* ^2^ statistics were used to evaluate heterogeneity: < 25% was interpreted as low, 25%–50% as moderate, 51%–75% as substantial and > 75% as considerable inconsistency. Substantial inconsistency decreases the quality of evidence by one level, and considerable inconsistency decreases the quality of evidence by two levels [22].

The GRADE pro system (https://www.gradepro.org) was used for each outcome from the meta‐analysis to create a summary of findings (Table 1).

**Table 1:** Grading of recommendations, assessment, development, and evaluation.

Quality assessment							No. of patients		Absolute (95% CI)	Quality	Importance
Studies	Study design	Risk of bias	Inconsistency	Indirectness	Imprecision	Other considerations	IG	CG			
**Apgar score at 1 min**											
*N* = 23 (1–23)	Randomized trials	Serious	Not serious	Not serious	Not serious	None	1763	1720	MD **0.11 SD higher** (0.05 higher to 0.18 higher)	XXXO moderate	Important
**Apgar score at 5 min**											
*N* = 24 (1–5, 7–25)	Randomized trials	Serious	Serious^a^	Not serious	Not serious	None	1772	1726	SMD **0.12 SD higher** (0 to 0.24 higher)	XXOO Low	Important
**Body birth weight**											
*N* = 51 (1–21, 23, 24, 26–53)	Randomized trials	Serious	Serious^b^	Not serious	Not serious	None	4022	4569	SMD **0.09 SD lower** (0.17 lower to 0.02 lower)	XXOO low	
**Body birth length**											
*N* = 20 (1–9, 18, 19, 21, 29, 31, 33, 41, 45, 48, 51, 52)	Randomized trials	Serious	Very serious^c^	Not serious	Not serious	None	1327	1764	SMD **0.01 SD lower** (0.28 lower to 0.29 higher)	XOOO very low	
**Head circumference at birth**											
*N* = 10 (1–7, 9, 31, 33)	Randomized trials	Serious	Not serious	Not serious	Not serious	None	845	1183	SMD **0.09 SD lower** (0.07 lower to 0.04 higher)	XOOO moderate	

*Note:* The symbols are indicated as follows: XOOO: very low quality of the evidence; XXOO: low quality of the evidence; XXXO: moderate quality of the evidence. Bold text refers to neonatal outcomes.

Abbreviations: CG, control group; CI, confidence interval; IG, intervention group; SMD, standardized mean difference.

^a^Inconsistency: The heterogeneity between studies was substantial (*I* ^2^ = 51*%*).

^b^Inconsistency: The heterogeneity between studies was substantial (*I* ^2^ = 61*%*).

^c^Inconsistency: The heterogeneity between studies was considerable (*I* ^2^ = 90*%*).

### 2.6. Statistical Analysis

A Microsoft Excel spreadsheet (Microsoft Corp., Redmond, Washington, United States) was utilized to compile and manage data extracted from the included studies. Statistical analyses, including the creation of forest plots, were performed using Review Manager (RevMan) Version 5.4.1. The Cochran Q statistic [23] was used to assess heterogeneity between studies. Heterogeneity, which reflects the variability in effect sizes (ESs) across studies, was assessed using the *I* ^2^ statistic.

AE, RE, CE, and overall exercise (i.e., AE, RE, and CE) effects on Apgar score, body composition, cardiorespiratory fitness, muscular fitness, motor fitness, and flexibility of the offspring were calculated, separately, for each study, following intervention and control groups′ coding of postinterventions and standard deviations (SDs). When we assessed offspring less than 1 month of age, we used the term “neonates,” whereas offspring older than 1 month, were called “children.” If we included both ages, then we referred them as “offspring.” Due to lack of evidence, cardiorespiratory fitness, muscular fitness, and flexibility were not assessed.

Secondary assessments were completed to analyze the influence of prenatal exercise during pregnancy (AE, RE, and CE) on neonatal Apgar score and body composition based on dichotomous maternal age (≤ 30 years or > 30 years) and pre‐pregnancy BMI classification (< 25 kg/m^2^, underweight/normal weight; 25–29.9 kg/m^2^, overweight; or ≥ 30 kg/m^2^, obese, respectively).

The standardized mean difference (SMD) was calculated based on differences in neonatal outcome values between groups. Data were required to be in one of the following forms: (a) mean and SDs, (b) 95% confidence interval (CI); or when this was unavailable, (c) actual *p* values for each group; or if only the level of statistical significance was available, (d) default *p* values (i.e., *p* < 0.05 becomes *p* < 0.049, *p* < 0.01 becomes *p* < 0.0099, and *p* when not significant becomes *p* > 0.05). A random‐effects model using the inverse variance method was applied to estimate the pooled SMD, with the ES estimated using the DerSimonian and Laird method [24]; under the assumption that the true effect across studies is randomly distributed around a central value. Forest plots were generated to demonstrate the study‐specific differences in posttraining neonatal outcome measures and their corresponding ESs within the respective 95% CIs. The study‐specific weight was derived as the inverse of the variance of the respective ESs, incorporating both within‐study variance and the between‐study variance component. The ESs of < 0.2, 0.2–0.5, 0.51–0.79, and ≥ 0.8 were considered trivial, small, moderate, and large, respectively [25].

The certainty of the evidence was further assessed using the GRADE framework. To enhance the rigor of the findings and address the potential influence of studies with a high risk of bias, advanced statistical procedures were performed using JASP (Version 0.18.3) and Jamovi (Version 2.6.45). A comprehensive casewise diagnostic analysis was performed to identify influential studies that could disproportionately bias the results. This involved calculating standardized residuals and Cook′s distance. When an influential study was identified, the SMD was recalculated both including and excluding the specific study to evaluate its impact on the stability and significance of the pooled estimates.

Potential publication bias was evaluated through visual inspection of funnel plots (Figure S1), complemented by Egger′s regression test to quantitatively assess funnel plot asymmetry.

## 3. Results

### 3.1. Study Selection

**Figure 1:**
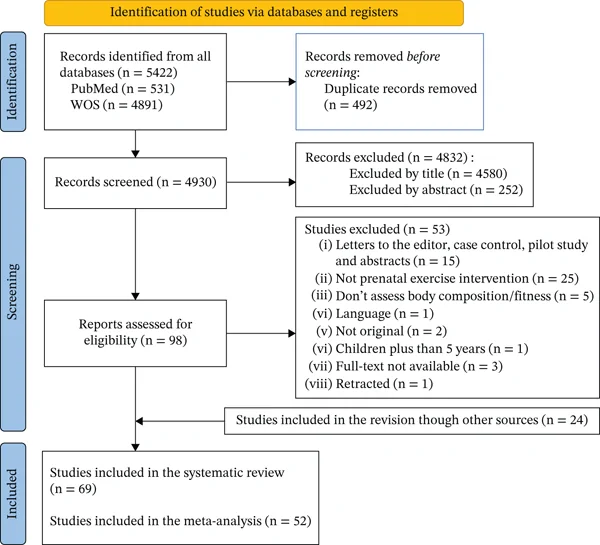
Flowchart of article selection process according to PRISMA.

The flow diagram according to PRISMA [16] shows that 5422 potentially relevant studies were found in PubMed and WOS databases. After 492 duplicates removed, 4930 studies were screened by title and abstract. Ninety‐eight studies were full‐text assessed for eligibility, of which 53 were excluded, which leaves 45 studies. However, at the end of selection process, 24 studies were manually included through other sources, thus leaving a total of 69 studies. In total, 69 studies were included in the systematic review and 52 in the meta‐analysis, the results are shown in Table S3. The reasons for excluding studies are shown in Figure 1.

### 3.2. Risk of Bias Assessment

**Figure 2:**
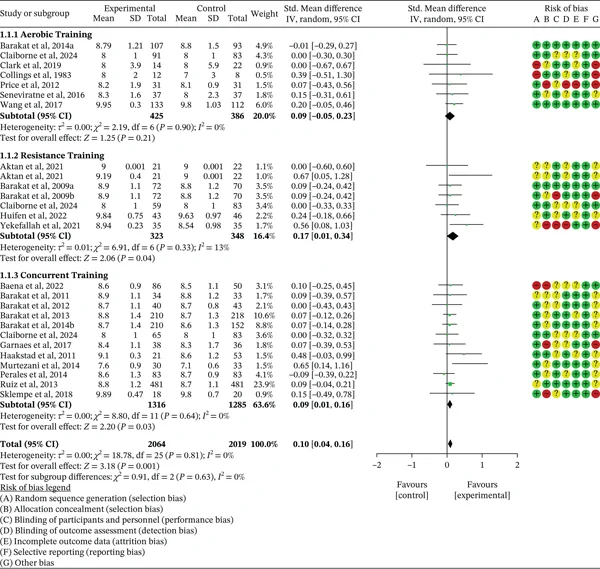
Forest‐plot of Apgar score at 1 min. Squares indicate the study‐specific effect size (ES) derived from comparison between children from exercise interventions and controls; horizontal lines indicate 95% confidence interval; diamonds indicate the summary ES estimate with its corresponding 95% confidence interval (size of diamonds reflects the study′s statistical weight).

**Figure 3:**
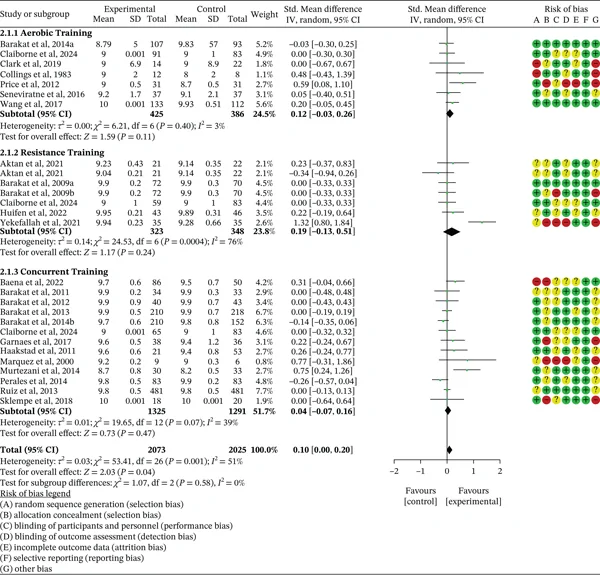
Forest‐plot of Apgar score at 5 min. Squares indicate the study‐specific effect size (ES) derived from comparison between children from exercise interventions and controls; horizontal lines indicate 95% confidence interval; diamonds indicate the summary ES estimate with its corresponding 95% confidence interval (size of diamonds reflects the study′s statistical weight).

**Figure 4:**
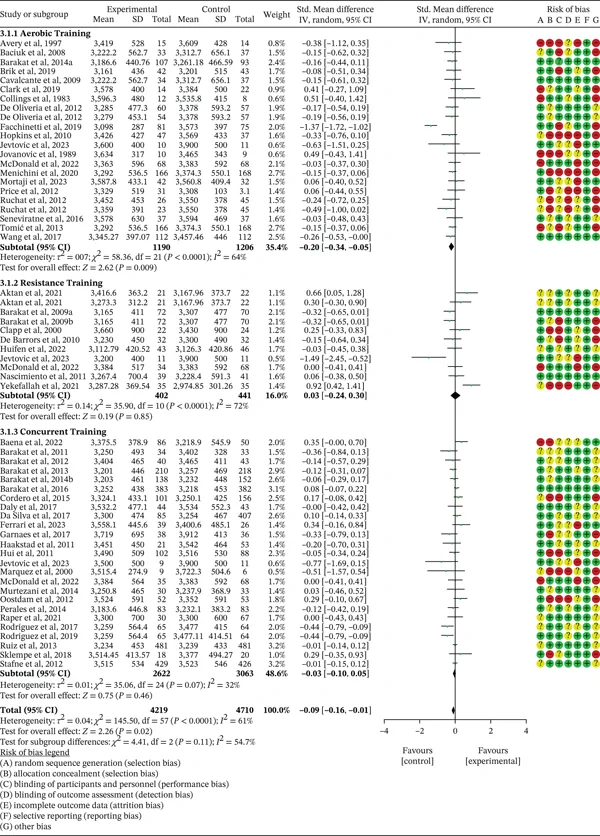
Forest‐plot of body birth weight. Squares indicate the study‐specific effect size (ES) derived from comparison between children from exercise interventions and controls; horizontal lines indicate 95% confidence interval; diamonds indicate the summary ES estimate with its corresponding 95% confidence interval (size of diamonds reflects the study′s statistical weight).

**Figure 5:**
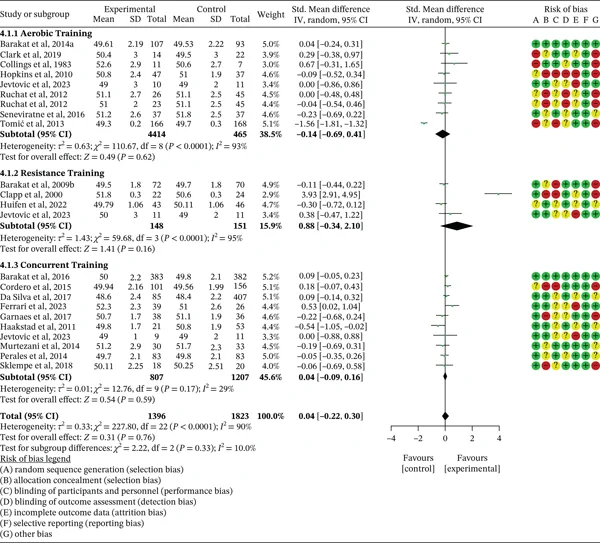
Forest‐plot of body birth length. Squares indicate the study‐specific effect size (ES) derived from comparison between children from exercise interventions and controls; horizontal lines indicate 95% confidence interval; diamonds indicate the summary ES estimate with its corresponding 95% confidence interval (size of diamonds reflects the study′s statistical weight).

**Figure 6:**
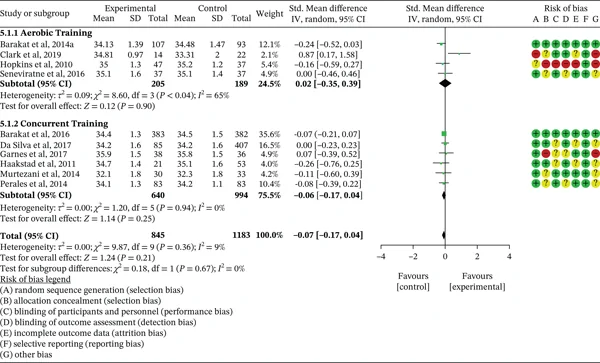
Forest‐plot of head circumference at birth. Squares indicate the study‐specific effect size (ES) derived from comparison between children from exercise interventions and controls; horizontal lines indicate 95% confidence interval; diamonds indicate the summary ES estimate with its corresponding 95% confidence interval (size of diamonds reflects the study′s statistical weight).

Reviewers (S.S‐P. and S.S‐B.) determined that 25 studies (36.23%) had some concerns, 35 studies (50.72%) were at a high risk and nine studies (13.04%) were at a low risk of bias across all domains. In the meta‐analysis (Figures 2, 3, 4, 5, and 6), 19 studies (36.54%) had some concerns, 27 studies (51.92%) were at high risk of bias and 6 studies (11.54%) were at low risk of bias. Missing outcome data was the most frequent reason for studies to be determined at high risk of bias both in the systematic review and in the meta‐analysis. For each outcome, the assessment of risk of bias is shown in Figures 2, 3, 4, 5, and 6.

### 3.3. Systematic Review

#### 3.3.1. Characteristics of Intervention

All 69 studies included an exercise intervention, and 13 studies included an intervention of exercise plus diet.

Most studies carried out exclusively an AE intervention. Four studies [26–29] combined AE plus stretching or pelvic floor exercises, and four studies executed a stretching/pelvic floor training alone [30–33]. Some researchers [34–37] carried out an exclusively RE intervention, whereas three studies [38–40] combined RE and stretching or pelvic floor training. Several studies performed CE and others combined CE and stretching or pelvic floor exercise. Three authors [41–43] divided their sample in a control group (that performed stretching) and three intervention groups (AE, RE, and CE, respectively), and Jevtovic et al. [44, 45] compared an intervention group (with women that performed AE, RE, and CE) with a control group (that performed stretching). Garshasbi and Faghih Zadeh [46] did not report the kind of exercise they performed in the intervention.

All studies reviewed completed their exercise interventions until at least the 34th gestational week. Twelve studies have no data about the duration of the interventions. Some studies have started their exercise interventions in the first trimester of pregnancy, the majority started in the second trimester and only four studies started them in the last trimester [34, 38, 39, 47].

The methodologies of the exercise interventions were heterogeneous. In relation to the frequency of training, most studies divided the exercise program across 3 days a week. Nine studies set a greater frequency of exercise sessions each week, whereas seven studies divided the exercise to once or twice per week. Some researchers set a variable weekly training frequency of 3–5 days, or 3–7 days.

Most exercise interventions were supervised, except eight studies that utilized unsupervised exercise interventions; eight studies combined supervised and nonsupervised exercise training sessions. Four studies did not provide data about whether the exercise intervention was supervised or not [33, 38, 48, 49].

The length of exercise sessions was between 30–60 min for almost all studies. Only two authors [39, 50] programed a longer session than 60 min. Clapp et al. [49] and Mortaji et al. [51] prescribed progressive sessions from 20 to 60 min, and 25 to 40 min, respectively; and two studies set a minimum of 15 min [33, 52] or 20 [48, 53, 54]. Bisson et al. [55] did not specify the length of the exercise sessions.

To monitor the intensity of the exercise sessions, the majority of included studies used percentage of maximum heart rate (%HRmax), rate of perceived exertion (RPE) [56] and/or percentage of maximum oxygen consumption (%VO_2_max); whereas 12 studies had no information about how exercise intensity was measured. The average maximum intensity for the interventions was 68.33% HRmax, RPE of 14.33, and 67.5%VO_2_max. The minimum and maximum intensity range values were between 30% [57] and 85% [58] of the HRmax, RPE from 10 [59] to 16 [52, 60] and 55% [48, 49]—80%VO_2_max [61].

In AE interventions, the majority of studies used RPE. Other studies monitored the intensity of training with %HRmax, %VO_2_max, percentage of peak oxygen consumption (%VO_2_peak), percentage of heart rate reserve (%HRR) and percentage of oxygen consumption reserve (%VO_2_R). The average maximum intensity for the interventions was 70.5% HRmax, RPE of 14.5, 65%VO_2_max, and 59%VO_2_peak. The minimum and maximum intensity range values were between 30% [57] and 80% [52] of the HRmax, RPE from 12 to 16 [52], 55% [48, 49]—75%VO_2_max [27] and from 30% [62] to 59% of the VO_2_peak.

In RE interventions, the majority of studies used and RPE. Other studies monitored the intensity of exercise training with RPE 0–10 [36], %VO_2_peak, %HRmax [35, 37, 39] and 1 repetition maximum [41, 43–45]. The average maximum intensity for the interventions was 71.6% HRmax and RPE of 14.7. The minimum and maximum intensity range values were between 60% [37, 39] and 80% [35] of HRmax and RPE between 12 [38, 41, 43] to 15 [41–43].

In CE interventions, the majority of studies used %HRmax and RPE. Other studies monitored the intensity of training with %VO_2_peak [41–43] and percentage of 1 repetition maximum [41, 43, 63, 64]. The average maximum intensity for the interventions was 68.5% HRmax and RPE of 14.33. The minimum and maximum intensity range values were between 55% [65, 66] and 85% [58] of HRmax and RPE from 10 [59] to 16 [60].

#### 3.3.2. Results of Interventions

None of the interventions presented a risk for maternal‐neonatal health. Most studies analyzed the effects of the exercise intervention on the neonates at birth or less than a month of age, except three studies that assessed outcomes when children were more than a month old [31, 51, 67].

Forty‐one of the 69 studies analyzed the effect of an exercise program during pregnancy on neonatal Apgar score at 1 and/or 5 min.

Three studies reported higher Apgar score at 1 and 5 min for neonates whose mothers were in the intervention group [39, 58, 68], and two other studies only found a higher score in Apgar score at 1 min, but not at 5 min [38, 69]. The other studies concluded that there were no significant differences between exercise‐exposed offspring and control offspring.

On one hand, at least one component of the HRPF was analyzed in all studies included in this review. All of them studied the effect of a prenatal exercise intervention in the offsprings′ body size (i.e., body weight, body length, head circumference…), 14 studies assessed adiposity of the offspring, and five of them measured their lean mass.

Seven studies reported lower body birth weight in neonates from women who attended prenatal exercise interventions relative to neonates whose mothers were controls.

Clapp et al. [48] observed an increased body birth weight in neonates whose mothers performed AE during pregnancy and McDonald et al. [42] reported that prenatal exercise predicted body birth weight. Six studies related lower risk of macrosomia in exercise intervention groups compared with controls. Hopkins et al. [70] concluded that neonates whose mothers trained during pregnancy had a lower BMI compared with controls. Claiborne et al. [41] found that neonates whose mothers performed CE during pregnancy presented a higher ponderal index and weight to length ratio (both, z‐score) than neonates of controls, as well as neonates of mothers that carried out AE presented a higher ponderal index than the neonates of controls. Haakstad et al. [69] observed a lower body birth length in neonates whose mothers did CE during pregnancy, whereas Ferrari et al. [50] found the opposite, and Clapp et al. [48] reported an increased body birth length associated with an AE intervention during pregnancy. Yang et al. [28] found that neonates of mothers that received personalized training containing AE and yoga had a lower body birth length compared with neonates of controls. Wang et al. [71], Menichini et al. [72], and Yang et al. [26] found a lower rate of large for gestational age (LGA) neonates, considered as the ones with a body birth weight at or above the 90th percentile for gestational age [73] in intervention groups compared with controls. Clark et al. [74] reported larger head circumference at birth in neonates of women who attended maternal exercise interventions relative to neonates of controls.

Two studies reported a decreased adiposity in neonates from the prenatal intervention group [12, 42]. Clapp et al. [49] concluded that children whose mothers decreased duration of exercise in the 2nd half of pregnancy had increased body birth weight, length, adiposity, ponderal index, head/abdominal ratio, fat mass relative to lean mass, and lean mass compared with those who maintained or increased the duration of exercise in the 2nd half of pregnancy.

No statistical differences from neonatal‐body composition outcomes were observed from the rest of studies analyzed, and none of the included studies assessed the effect of prenatal exercise on cardiorespiratory fitness, muscular fitness, or flexibility of the children up to 5 years of age. Two studies assessed the motor fitness of the children [31, 75].

McMillan et al. [75] showed a positive influence of prenatal AE in the locomotion skills of the neonates at 1 month (*p* = 0.02) compared with neonates not exposed to exercise in utero. Hellenes et al. [31] concluded that boys but not girls in the intervention group had lower fine motor skills compared with children of controls (95% CI: 10.3–11.0 vs. 11.0–11.9; *p* = 0.01).

### 3.4. Meta‐Analysis

#### 3.4.1. Characteristics of Intervention and Outcomes

In this meta‐analysis, all studies included exercise interventions (AE, RE, and/or CE) and control groups continued with normal health care in most of them.

The present meta‐analysis assessed 22 AE interventions from 20 studies, 9 RE interventions from eight studies, and 24 CE interventions from 24 studies. Three authors [41–43] performed three exercise interventions (AE, RE, and CE). Similarly, de Oliveria et al. [52] and Ruchat et al. [57] performed two different AE interventions, whereas Aktan et al. [38] performed two RE interventions.

To carry out this meta‐analysis, we identified the five most common outcomes assessed (Apgar score at 1 and 5 min, body birth weight, body birth length, and head circumference at birth).

Results of Apgar score were obtained by medical health records for all studies, except four [34, 38, 51, 76] that did not state how the measurements were obtained.

The most widely used device for measuring body birth weight was medical health records, followed by electronic scale [48, 52, 70, 77], except in two studies [39, 63] that were self‐reported. Twelve studies did not specify the measuring tool used.

To assess body birth length, all studies used medical health records, except Hopkins et al. [70], who utilized a nanometer, and Clapp et al. [48], who used a tape measure. Two authors [34, 51] did not specify the measurement tool used.

Results of head circumference at birth were obtained by medical health records, except three studies [34, 38, 76] that did not indicate the measuring device used.

#### 3.4.2. Effects of Exercise Training During Pregnancy on Apgar Score at 1 min

Outcomes for Apgar score at 1 min are shown in Figure 2. The Apgar score at 1 min measurement was separated into AE, RE, and CE subgroup analyses due to potential heterogeneity of results.

The pooled mean ES estimated of comprised groups of AE intervention outcomes was assessed from seven studies, RE interventions from six studies (with seven intervention groups), and CE interventions from 12 studies.

The SMD of 0.09 points (*p* = 0.21; *I* ^2^ = 0*%*) indicated a small effect in favor of AE with no significant differences between groups. For the analysis of RE, a SMD of 0.17 points (*p* = 0.04; *I* ^2^ = 13*%*) indicated a trivial effect in favor of RE with a significant difference between groups. In CE, the SMD of 0.09 points (*p* = 0.03; *I* ^2^ = 0*%*) indicated a trivial effect in favor of CE with a significant difference between groups.

In addition, the effect of AE, RE, and CE on body birth weight was 0.10 points (*p* = 0.001; *I* ^2^ = 0*%*) and the level of evidence according to the GRADE rating was moderate (Table 1). Sensitivity analysis for this outcome identified Ruiz et al. [78] as a highly influential study through casewise diagnostics. However, the pooled ES remained stable and did not significantly change (original SMD = 0.10, 95% CI: 0.04–0.16; recalculated SMD = 0.09, 95% CI: 0.03–0.18), indicating that the primary results are robust and not disproportionately driven by any single study with high risk of bias confirming the stability of the finding.

When we assessed the influence of prenatal exercise on Apgar score at 1 min according to maternal age and pre‐pregnancy BMI (Table S4), results indicated that neonates whose mothers (at any age) that carried out overall exercise during pregnancy had a higher Apgar score at 1 min compared with controls (ES = 0.18; *p* = 0.03; *I* ^2^ = 0*%*, and ES = 0.09; *p* = 0.03; *I* ^2^ = 0*%*, respectively), and neonates whose mothers were older than 30 years and performed CE presented higher Apgar score at 1 min compared with controls (ES = 0.09; *p* = 0.03; *I* ^2^ = 0*%*). However, when we assessed AE and RE separately, no significant differences were found between maternal age groups. Regarding pre‐pregnancy BMI, neonates whose mothers were at normal weight and performed overall exercise had higher Apgar score at 1 min (ES = 0.09; *p* = 0.01; *I* ^2^ = 0*%*). There is also a tendency for a better Apgar score at 1 min of neonates with normal weight mothers who did RE (ES = 0.19; *p* = 0.05; *I* ^2^ = 2*%*). No significant differences were obtained from the rest of the variables assessed (Table S4).

#### 3.4.3. Effects of Exercise Training During Pregnancy on Apgar Score at 5 min

Outcomes for Apgar score at 5 min are shown in Figure 3. Apgar score at 5 min was separated into AE, RE, and CE subgroup analyses due to potential heterogeneity of results.

The pooled mean ES estimated of comprised groups of AE intervention outcomes was assessed from seven studies, RE interventions from six studies (with seven intervention groups), and CE interventions from 13 studies.

The SMD of 0.12 points (*p* = 0.11; *I* ^2^ = 3*%*) indicated a trivial effect in favor of AE with no significant differences between groups. For the analysis of RE, a SMD of 0.19 points (*p* = 0.24; *I* ^2^ = 76*%*) indicated a trivial effect in favor of RE with no significant differences between groups. In CE, the SMD of 0.04 points (*p* = 0.47; *I* ^2^ = 39*%*) indicated a trivial effect in favor of CE with no significant differences between groups.

In addition, the effect of AE, RE, and CE on Apgar score at 5 min was 0.10 points (*p* = 0.04; *I* ^2^ = 51*%*) and the level of evidence according to the GRADE rating was low (Table 1). Sensitivity analysis for this outcome identified Garnaes et al. [77] as a highly influential study through casewise diagnostics. However, the pooled ES remained stable and did not significantly change (original SMD = 0.10, 95% CI: 0.00–0.20; recalculated SMD = 0.10, 95% CI: −0.00 to 0.20), indicating that the primary results are robust and not disproportionately driven by any single study with high risk of bias confirming the stability of the finding.

When we assessed the influence of prenatal exercise on Apgar score at 5 min according to maternal age and pre‐pregnancy BMI, results indicated that regarding maternal age, only neonates whose mothers were younger than 30 years and performed AE during pregnancy had neonates with higher Apgar score at 5 min relative to controls (ES = 0.39; *p* = 0.04; *I* ^2^ = 0*%*). No significant differences were obtained from the rest of the variables assessed (Table S4).

#### 3.4.4. Effects of Exercise Training During Pregnancy on Body Birth Weight

Outcomes for body birth weight are shown in Figure 4. Body birth weight outcome was separated into AE, RE, and CE subgroup analyses due to potential heterogeneity of results.

The pooled mean ES estimated of comprised groups of AE intervention outcomes was assessed from 21 studies (23 interventions), RE interventions from 10 studies (11 interventions), and CE interventions from 25 studies.

The SMD of −0.20 points (*p* = 0.009; *I* ^2^ = 64*%*) indicated a small effect in favor of AE with a significant difference between groups. For the analysis of RE, a SMD of 0.03 points (*p* = 0.85; *I* ^2^ = 72*%*) indicated a trivial effect in favor of RE with no significant differences between groups. In CE, the SMD of −0.03 points (*p* = 0.46; *I* ^2^ = 32*%*) indicated a trivial effect in favor of CE with no significant differences between groups.

In addition, the effect of AE, RE, and CE on body birth weight was −0.09 points (*p* = 0.02; *I* ^2^ = 61*%*) and the level of evidence according to the GRADE rating was low (Table 1). Sensitivity analysis for this outcome identified Facchinetti et al. [47]; Barakat et al. [64]; Ruiz et al. [78] and Stafne et al. [79] as a highly influential study through casewise diagnostics. However, the pooled ES remained stable and did not significantly change (original SMD = −0.09, 95% CI: −0.16 to −0.01; recalculated SMD without Facchinetti et al. [47] = −0.06, 95% CI: −0.12 to 0.05; without Barakat et al. [64] = −0.09, 95% CI: −0.17 to −0.01; without Ruiz et al. [78] = −0.09, 95% CI: −0.17 to −0.01; without Stafne et al. [79] = −0.09, 95% CI: −0.17 to 0.05), indicating that the primary results are robust and not disproportionately driven by any single study with high risk of bias confirming the stability of the finding.

When we assessed the influence of prenatal exercise on body birth weight according to maternal age (≤ 30 years vs. > 30 years) and pre‐pregnancy BMI (underweight/normal weight, overweight, and obese), results indicated that women younger than 30 years who exercised during pregnancy did not have significant differences in body birth weight compared with controls. However, pregnant women older than 30 years had lower neonatal body birth weight within normal values in overall exercise (ES = −0.10; *p* = 0.03; *I* ^2^ = 64*%*) and AE (ES = −0.28; *p* = 0.02; *I* ^2^ = 77*%*). No significant differences were obtained in RE and CE. Similarly, results demonstrated lower body birth weight within normal values in normal weight women who performed overall exercise (ES = −0.09; *p* = 0.04; *I* ^2^ = 47*%*) and AE (ES = −0.17; *p* = 0.006; *I* ^2^ = 0*%*) during pregnancy. No significant differences were found in RE and CE, or in overweight and obese women regarding birth weight outcomes (Table S4).

#### 3.4.5. Effects of Exercise Training During Pregnancy on Body Birth Length

Outcomes for body birth length are shown in Figure 5. Body birth length outcome was separated into AE, RE, and CE subgroup analyses due to potential heterogeneity of results.

The pooled mean ES estimated of comprised groups of AE intervention outcomes was assessed from nine studies (with 10 intervention groups), RE interventions from three studies [34, 35, 43], and CE interventions from 10 studies.

The SMD of −0.14 points (*p* = 0.62; *I* ^2^ = 93*%*) indicated a trivial effect in favor of AE with no significant differences between groups. For the analysis of RE, a SMD of 0.88 points (*p* = 0.16; *I* ^2^ = 95*%*), which indicated a large effect in favor of RE with no significant differences between groups. In CE, the SMD of 0.04 points (*p* = 0.59; *I* ^2^ = 29*%*) indicated a trivial effect in favor of CE with no significant differences between groups.

In addition, the effects of AE, RE, and CE on body birth length were 0.04 points (*p* = 0.76; *I* ^2^ = 90*%*) and the level of evidence according to the GRADE rating was very low (Table 1). Sensitivity analysis for this outcome identified Tomic et al. [80] and Clapp et al. [48] as highly influential studies through casewise diagnostics. However, the pooled ES remained stable and did not significantly change (original SMD = 0.04, 95% CI: −0.22 to 0.30; recalculated SMD without Tomic et al. [80] = 0.08, 95% CI: −0.09 to 0.25; recalculated SMD without Clapp et al. [48] = −0.08, 95% CI: −0.31 to 0.15), indicating that the primary results are robust and not disproportionately driven by any single study with high risk of bias confirming the stability of the finding.

When we assessed the influence of prenatal exercise on body birth length according to maternal age and pre‐pregnancy BMI, no significant differences were found between maternal age or pre‐pregnancy BMI groups (Table S4).

#### 3.4.6. Effects of Exercise Training During Pregnancy on Head Circumference at Birth

Outcomes for head circumference at birth are shown in Figure 6. Outcomes for head circumference at birth were separated into AE and CE subgroup analyses due to potential heterogeneity of results.

The pooled mean ES estimated of comprised groups of AE intervention outcomes was assessed from four studies [29, 70, 74, 81] and CE interventions from six studies.

The SMD of 0.02 points (*p* = 0.90; *I* ^2^ = 65*%*) indicated a trivial effect in favor of AE with no significant differences between groups. In CE, the SMD of −0.06 points (*p* = 0.25; *I* ^2^ = 0*%*) indicated a trivial effect in favor of CE with no significant differences between groups.

In addition, the effect of AE, RE, and CE on head circumference at birth was −0.07 points (*p* = 0.21; *I* ^2^ = 9*%*) and the level of evidence according to the GRADE rating was moderate (Table 1). Sensitivity analysis for this outcome identified Barakat et al. [64] as a highly influential study through casewise diagnostics. However, the pooled ES remained stable and did not significantly change (original SMD = −0.07, 95% CI: −0.17 to 0.04; recalculated SMD = −0.06, 95% CI: −0.20 to 0.08), indicating that the primary results are robust and not disproportionately driven by any single study with high risk of bias confirming the stability of the finding.

When we assessed the influence of prenatal exercise on head circumference at birth according to maternal age and pre‐pregnancy BMI, results indicated that neonates whose mothers were younger than 30 years old and performed AE interventions had a greater head circumference at birth compared with controls (*E* *S* = 0.87; *p* = 0.02; *I* ^2^ not applicable). However, women older than 30 years who performed overall exercise during pregnancy had neonates with smaller head circumferences at birth than controls (*E* *S* = −0.11; *p* = 0.04; *I* ^2^ = 0*%*). No significant differences between groups were found in the rest of variables assessed regarding maternal age and pre‐pregnancy BMI (Table S4).

#### 3.4.7. Publication Bias

Visual inspection of the funnel plots generated in JASP and Jamovi showed a generally symmetrical distribution (Figure S1). This observation was statistically supported by Egger′s regression test (Table S5), which yielded no evidence of significant publication bias for Apgar score at 1 min (*p* = 0.113), birth weight (*p* = 0.906), and head circumference (*p* = 0.327). Although significant funnel plot asymmetry was detected for Apgar score at 5 min (*p* = 0.008) and birth length (*p* = 0.003), the robustness of these findings was subsequently confirmed through leave‐one‐out sensitivity analyses and casewise diagnostics.

## 4. Discussion

The present systematic review with meta‐analysis evaluated experimental studies with exercise interventions in pregnant women and their effects on offspring Apgar score and HRPF up to 5 years of age. The findings showed that prenatal exercise improves Apgar score at 1 and 5 min and reduces body birth weight within normal values in neonates.

The systematic review included only studies analyzing the influence of exercise during pregnancy on offspring Apgar score, body composition, and motor fitness. None of the included studies assessed the effect of prenatal exercise on cardiorespiratory fitness, muscular fitness, or flexibility of the children up to 5 years of age.

Regarding the meta‐analysis, we identified the five most common outcomes assessed (i.e., Apgar score at 1 and 5 min, body birth weight, body birth length, and head circumference at birth). There is a gap in the literature in reference to the effect of exercise during pregnancy in all components of offspring HRPF except for body composition. Nevertheless, there is still a lack of evidence concerning adiposity, lean mass, and bone density of offspring whose mothers exercised during pregnancy.

As a result, this systematic review with meta‐analysis evaluated the influence of exercise during pregnancy on Apgar score, body composition, and motor fitness up to 5 years old. When the findings of the systematic review match the findings from the meta‐analysis, only the last ones will be commented. Otherwise, we will specify the difference between the analyses. Data regarding the outcomes that were not included in the meta‐analysis are based on the systematic review.

### 4.1. Effects of Exercise Training During Pregnancy on Apgar Score

Our results indicate that children whose mothers performed overall exercise, RE, and CE interventions during pregnancy, but not AE, have higher Apgar score at 1 min after birth. RE showed the biggest effect, but when we assessed the sample by maternal age and pre‐pregnancy BMI, significant differences were found only in the neonates whose mothers performed CE and were older than 30 years. Overall exercise presented significant differences in both groups of maternal age. Although it seems that increasing pre‐pregnancy BMI decreases the influence of exercise on Apgar score, it could be explained by the insufficient number of studies with an obese sample of participants.

For Apgar score at 5 min, the forest plot shows that neonates whose mothers did exercise interventions have a higher Apgar score than the neonates of controls. Nevertheless, these results should be interpreted with caution, as publication bias has been detected in this outcome, as confirmed by Egger′s regression test. Additionally, when we assessed AE, RE, and CE interventions individually, none of them reported significant differences between groups. Regarding maternal age and pre‐pregnancy BMI, only AE interventions tend to improve Apgar score at 5 min when women were younger than 30 years.

Along with the effect of exercise during pregnancy on body composition, Apgar score is the second outcome most commonly assessed by evidence in this systematic review with meta‐analysis.

In our meta‐analysis, it seems that exercise during pregnancy has a major impact on how the neonate tolerates the birthing process (Apgar score at 1 min). This could potentially be explained by the influence of exercise during pregnancy and maternal fitness in reducing the risk of instrumental birth and cesarean section [82, 83]. However, the influence of prenatal exercise on Apgar score is not clear based on the literature [84–86], which could be explained by the numerous factors that can influence this outcome, including maternal sedation or anesthesia, congenital malformations, gestational age, trauma and interobserver variability. In addition, several elements assessed by the Apgar score can be subjective [87] and alone cannot predict individual neonatal mortality or neurologic outcomes [87].

### 4.2. Effects of Exercise Training During Pregnancy on Body Composition

#### 4.2.1. Effects of Exercise Training During Pregnancy on Body Birth Weight

In our meta‐analysis, results indicate that overall exercise and AE interventions during pregnancy reduce neonatal body birth weight within the normal range. When we assessed the sample by maternal age and pre‐pregnancy BMI, overall exercise and AE during pregnancy only presented significant differences between groups in body birth weight when women were older than 30 years and with normal weight, possibly due to a lack of studies assessing this outcome with obese and older pregnant women.

Previous reviews of prenatal exercise have evaluated randomized controlled clinical trials assessing the influence of exercise interventions (without distinguishing between AE, RE, and CE) during pregnancy on body birth weight and support that structured exercise programs during pregnancy contribute to maintain the body birth weight within normal values [84, 86, 88], which is between low body birth weight (< 2500 g) and macrosomia (> 4000 g) [64, 89]. Although macrosomia and LGA terms have been used interchangeably, LGA describes a neonate whose body birth weight is at or above the 90th percentile for gestational age [73]. In this context, the latest published reviews support that exercise interventions (without distinguishing between AE, RE, and CE) during pregnancy decrease the number of LGA neonates, regardless of pre‐pregnancy BMI of the mother [90–92].

Physical inactivity is linked to perinatal mortality and morbidity, lower Apgar score, future cardiovascular diseases, Type 2 diabetes and obesity. However, pregnant women are sometimes advised to reduce physical activity levels due to the belief that it could adversely affect fetal development, reduce fetal growth and body birth weight [93]. Our meta‐analysis supports that exercise in pregnancy results in a modest decrease in body birth weight within the normal range, which could lead to favorable health outcomes as a long‐term reduction in the risk of obesity later in life [94, 95]. In addition, Bhargava et al. [96] reported that the obesogenic factor attributed to a low body birth weight could be explained by a fast weight gain of these children fed by their parents to catch up growth.

On the other hand, McEwen et al. [97] found out that neonates between the 50th–93rd percentiles in body birth weight had lower levels of perinatal mortality and higher scores in education outcomes. Jeon et al. [98] have reported a minor perinatal mortality in neonates with a body birth weight between 3491–3890 g.

The mechanisms underlying the effects of exercise on fetal growth remain unclear, and authors report limitations in the methodology of the interventions due to their heterogeneity [70]. In our meta‐analysis, substantial heterogeneity was detected and low quality of evidence according to the GRADE rating, which could present an increased risk of bias. Although the most widely used device for measuring body birth weight was medical health records, as other researchers have reported [99], there is a need for a standardized tool to assess this neonatal outcome.

#### 4.2.2. Effects of Exercise Training During Pregnancy on Body Birth Length

There were no significant differences regarding prenatal exercise and body birth length in our meta‐analysis, regardless of the intervention used and the maternal age or pre‐pregnancy BMI. Indeed, the studies, which reported the highest SMDs between groups were categorized with high risk of bias [48, 80].

Krebs et al. [100, 101] reported that body birth length is the strongest predictor of linear growth status and development in the first 2 years of life. Indeed, high values of body birth length have been related to an increased risk of hypertension in childhood [102]. Maehle et al. [103] observed a higher risk of mortality in breast cancer patients whose body birth length was greater than 48 cm, hypothesizing that the insulin‐like growth factor system could be involved in the underlying mechanisms.

In other studies, not included in the meta‐analysis due to the no‐compliance of the inclusion criteria, no differences were found in body birth length of neonates exposed or not exposed to gestational exercise [104–106]. However, Juhl et al. [107] described a decreased body birth length with increasing maternal self‐reported exercise when controlling for confounding variables such as maternal BMI, age, and smoking habit.

#### 4.2.3. Effects of Exercise Training During Pregnancy on Head Circumference at Birth

No significant differences were found between head circumference at birth of children whose mothers exercised during pregnancy and the ones whose mothers were controls. When we assessed the influence of prenatal exercise on head circumference at birth by maternal age and pre‐pregnancy BMI subgroups, we observed a trivial decrease in head circumference of neonates whose mothers were older than 30 years and performed exercise during pregnancy. We also observed a large effect of AE during pregnancy in women younger than 30 years on head circumference at birth. Nevertheless, this result is verified only by one study, categorized with high risk of bias; thus we need further evidence to confirm this issue.

In our meta‐analysis, only Clark et al. [74] reported larger head circumference at birth in neonates of the women who performed maternal exercise interventions. Indeed, these authors conclude that neonatal head circumference is predicted by prenatal AE levels. In this context, Qian et al. [108] suggested that neonatal head circumference at birth was positively associated to intelligence, which might indicate that brain development could be responsible for the development of intelligence. These results are in agreement with some researchers who found that prenatal brain growth may be causally related to brain volume, neurodevelopment, and childhood academic performance [109–111].

#### 4.2.4. Effects of Exercise Training During Pregnancy on Offspring Adiposity

McDonald et al. [12] reported a minor adiposity in children from the prenatal exercise intervention group. Previous reviews showed that prenatal exercise resulted in the delivery of lighter and leaner neonates at birth [112, 113]. The evidence supports that adipose tissue at birth is predictive of the risk of overweight or obesity in childhood, adolescence, and adulthood. These hypotheses strongly support maternal exercise interventions during the initial development of fetal adipose [114, 115].

An important limitation of this outcome is the lack of a standardized tool to assess adiposity and lean mass of the children. Although two studies [12, 62] measured adiposity with estimation equations from the biceps, triceps, and subscapular skinfolds [116], Clapp et al. [48, 71] used equations with five skinfolds [117]. Garnaes et al. [77] used only the skinfold thickness values in millimeters, and other studies assessed adiposity with abdominal and/or hip circumferences [61, 62, 67, 74] or whole‐body dual‐energy x‐ray absorptiometry [49, 67, 70]. Thus, none of the assessments was the same, from birth [61, 77] to 1 year [67], which makes comparison of the studies in a meta‐analysis impossible.

### 4.3. Effects of Exercise Training During Pregnancy on Motor Fitness

Only three studies assessed the influence of exercise during pregnancy on motor fitness of the children [31, 51, 75] with inconclusive results. McMillan et al. [75] showed a positive influence of prenatal exercise in the locomotion skills of the children at 1 month. The findings of this study are consistent with previously reported positive effects on neuromotor skills in children of 5 years whose mothers continued practicing AE, compared with the ones that were active but stopped exercise during gestation [15]. However, Hellenes et al. [31] concluded that 18 month old boys, but not girls, whose mothers were in the CE intervention group, had lower fine motor skills than controls. These differences in findings might be explained by the insufficient frequency of training (less than 3 times per week) and the lack of information about the type of exercise performed in the intervention [75]. Mortaji et al. [51] reported no differences between children from exercise and control groups. The ACOG [14] has published recommendations for training during pregnancy to get the greatest benefits to maternal health: at least 3 or 4 days a week of CE, at an intensity less than 60%–80% of maximum heart rate (HRmax) or 12–14 RPE, from the first trimester to birth, in 30 to 60‐min sessions.

Several studies have reported that boys are more susceptible to developing coordination disorders than girls [118, 119]. This sex difference is present in the intrauterine period; potentially male fetuses are more vulnerable to impairment than females [120]. Sex differences in motor fitness could be presented already in the intrauterine environment. As exercise could affect the early programing of the fetus, prenatal exercise could impact children’′s motor fitness; however, more studies analyzing fetal sex differences are needed to understand the mechanisms involved in motor skills of children. In addition, the lack of evidence regarding the association between prenatal exercise and motor fitness of children makes a need for future longitudinal experimental studies which assess the impact of exercise during pregnancy on motor fitness in childhood.

### 4.4. Strengths and Limitations

The main strength of this study is that this is the first systematic review with meta‐analysis, which assesses the effect of exercise interventions during pregnancy on all domains of the Apgar score and HRPF of the offspring up to 5 years. Another strength would be the assessment of the influence of AE, RE, and CE, separately, and by maternal BMI and age groups.

A possible limitation of our systematic review is the considerable heterogeneity in the methodological design of the interventions included, in terms of length of interventions, type of exercise, as well as frequency, duration, intensity, and supervision of the exercise sessions. Other confounding factors, such as maternal and child lifestyle behaviors (nutrition, smoking, alcohol, and socioeconomic status) may also increase data variations. Additionally, the study outcomes were not always assessed in a standardized method.

Finally, the adherence of exercise interventions was not assessed or reported in 40% of the included studies, which represents a high risk of bias.

## 5. Conclusions

Overall, Apgar score at 1 min was higher in neonates whose mothers performed prenatal overall exercise, RE, and CE, but not AE. Neonates from exercise intervention groups showed a higher Apgar score at 5 min, but not when we assessed the effect of AE, RE, and CE interventions separately. Moreover, prenatal exercise and AE interventions, but not CE and RE, seem to reduce body birth weight within normal values. Notwithstanding, there were no significant differences in birth length irrespective of the intervention used. Moreover, there is a lack of evidence regarding the influence of prenatal exercise on cardiorespiratory fitness, muscular fitness, flexibility, and motor fitness of children.

Further follow‐up studies assessing the influence of exercise interventions during pregnancy on children′s HRFP are needed. To guarantee the consistency and comparability of the studies, a standardized methodology of exercise interventions is also required.

## Author Contributions

Sandra Sánchez‐Parente: guarantor, conceptualization, methodology, data curation, formal analysis, writing—original draft, writing—review and editing, led the study design and writing, and contributed to the acquisition, analysis, and interpretation of data. Dr. Ángela Rodríguez‐Perea: methodology, formal analysis, writing—review and editing, and led the data analysis. Sandra Solaguren‐Beaskoa: data curation, formal analysis, and contributed to data acquisition and analysis. Dr. Laura Baena‐García: writing—review and editing and critically reviewed and revised the manuscript. Prof. Linda E. May: writing—review and editing and critically reviewed and revised the manuscript. Prof. José Castro‐Piñero: conceptualization, methodology, data curation, formal analysis, and contributed to study design, data acquisition, and analysis and interpretation. Prof. Virginia A. Aparicio: conceptualization, methodology, data curation, formal analysis, and contributed to study design, data acquisition, and analysis and interpretation. All authors: writing—review and editing, supervision, validation, approval of final version. Sandra Sánchez‐Parente and Ángela Rodríguez‐Perea contributed equally to this work.

## Funding

This study was supported by the Andalusian FEDER operational program (B‐cts‐162‐ugr18); FP7 People: Marie‐Curie Actions (10.13039/100011264, COFUND—Agreement n°291780); Ministry of Economy, Innovation, Science, and Employment of the Board from Andalusia (COFUND—Agreement n°291780); National Plan for Scientific and Technical Research and Innovation 2017–2020 (PN/FPU20/02938).

## Disclosure

All authors reviewed and discussed the results, contributed to the final manuscript, agreed on the order of authorship, and approved the final version.

## Conflicts of Interest

The authors declare no conflicts of interest.

## Supporting Information

Additional supporting information can be found online in the Supporting Information section.

## Supporting information


**Supporting Information 1** Table S1 shows the search strategy utilized in the electronic databases. Table S2 shows the mesh terms used in the search strategy and their entry terms. Table S3 shows the main characteristics of the studies included in the systematic review. Table S4 shows the influence on neonatal outcomes by maternal age and pre‐pregnancy body mass index. Table S5 shows the publication bias of the neonatal outcomes. Figure S1 shows the funnel‐plot of neonatal outcomes (Apgar score at 1 min, Apgar score at 5 min, body birth weight, body birth length, and head circumference at birth). Figure S1.1 shows the funnel‐plot of Apgar score at 1 min. Figure S1.2 shows the funnel‐plot of Apgar score at 5 min. Figure S1.3 shows the funnel‐plot of body birth weight. Figure S1.4 shows the funnel‐plot of body birth length. Figure S1.5 shows the funnel‐plot of head circumference at birth.

**Supporting Information 2**  

## Data Availability

Data sharing is not applicable to this article as no datasets were generated or analyzed during the current study.
