# Supplementary material for: Influence of Prenatal Exercise on Apgar Score and Health‐Related Physical Fitness in Childhood: A Systematic Review and Meta‐Analysis
Source: J Pregnancy. 2026 Sep 3;2026:6702095. doi: 10.1155/jp/6702095 (PMC13542584; doi:10.1155/jp/6702095)
Supplement: Supplementary file 1 [file JP-2026-6702095-s001.pdf]

Table S1. Search strategy utilized in the electronic databases.

Database	*Search*	*Filters*
PubMed	((“Pregnant women”[Mesh] OR “Pregnancy”[Mesh] OR “pregnant”[All Fields] OR “Mother-Child Relations”[Mesh] OR “Maternal-Fetal Relations”[Mesh]) AND (“Exercise”[Mesh])) AND ((“Infant”[Mesh] OR “child”[Mesh] OR “Child, Preschool”[Mesh] OR “Offspring”[All Fields]) AND (“Physical Fitness”[Mesh] OR “Cardiorespiratory Fitness”[Mesh] OR “Physical Conditioning, Human”[Mesh] OR “Motor Skills”[Mesh] OR “Psychomotor Performance”[Mesh] OR “Motor development”[All Fields] OR "Muscle Strength"[Mesh] OR "Muscle strength dynamometer"[Mesh] OR “Range of motion, articular”[Mesh Major Topic] OR "Postural Balance"[MeSH] OR “Cardiovascular fitness”[All Fields] OR “Aerobic fitness”[All Fields] OR “Aerobic capacity”[All Fields] OR “Maximal oxygen consumption”[All Fields] OR “VO2max”[All Fields] OR “Motor fitness"[All Fields] OR "Running Speed"[All Fields] OR “Agility”[All Fields] OR “Anthropometry”[Mesh] OR “Body Composition”[Mesh] OR “Body Constitution”[Mesh] OR “Body Weights and Measures”[Mesh] OR “Body Size”[Mesh] OR “Body Weight”[Mesh] OR “Weight”[All Fields] OR “Waist Circumference”[Mesh] OR “Neck circumference”[All Fields] OR “Skinfold Thickness”[Mesh] OR “Waist-Hip Ratio”[Mesh] OR “DEXA”[All Fields] OR “Fetal Macrosomia”[All Fields] OR “Birth Weight”[Mesh]))	Publication date: “until 10/12/2024” Species: “humans” Languages: “English” or “Spanish”
WOS	((“Pregnant women” OR “Pregnant Woman” OR “Pregnancy” OR “Pregnancies” OR “Gestation” or “Pregnant” OR “Mother-Child Relations” OR “Mother Child Relations” OR “Mother-Child Relation” OR “Mother-Child Interaction” OR “Mother Child Interaction” OR “Mother-Child Interactions” OR “Mother-Child Relationship” OR “Mother Child Relationship” OR “Mother-Child Relationships” OR “Mother-Infant Interaction” OR “Mother Infant Interaction” OR “Mother-Infant Interactions” OR “Mother-Infant Relations” OR “Mother Infant Relations” OR “Mother-Infant Relation” OR “Maternal-Fetal Relations” OR “Maternal Fetal Relations” OR “Mother-Fetus Relationship” OR “Mother Fetus Relationship” OR “Mother-Fetus Relationships” OR “Mother-Fetus Relations” OR “Mother Fetus Relations”) AND (“Exercise” OR “Exercises” OR “Physical Activity” OR “Physical Activities” OR “Physical Exercise” OR “Physical Exercises” OR “Acute Exercise” OR “Acute Exercises” OR “Isometric Exercises” OR “Isometric Exercise” OR “Aerobic Exercise” OR “Aerobic Exercises” OR “Exercise Training” OR “Exercise Trainings”)) AND ((“Infant” OR “child” OR “children” OR “Child, Preschool” OR “Preschool Child” OR “Preschool Children” OR “offspring”) AND ("Muscle strength" OR "Muscular strength" OR dynamometer OR “Joint Range of Motion” OR “Joint flexibility” OR “Range of motion” OR “Musculoskeletal Equilibrium” OR “Postural Balance” OR “Postural Equilibrium” OR “Cardiorespiratory fitness” OR “Cardiovascular fitness” OR “Aerobic fitness” OR “Aerobic capacity” OR “Maximal oxygen consumption” OR “VO2max” OR "Physical Endurance" OR “Running Speed” OR “Agility” OR "Motor fitness" OR “Physical Fitness” OR “Physical Conditioning” OR “Motor Skills” OR “Human Physical Conditioning” OR “Motor Skill” OR “Psychomotor Performance” OR “Psychomotor Performances” OR “Visual Motor Coordination” OR “Visual Motor Coordination” OR “Perceptual Motor Performance” OR “Perceptual Motor Performances” OR “Sensory Motor Performance” OR “Sensory Motor Performances” OR “Motor development” OR “Anthropometry” OR “Body Composition” OR “Body Constitution” OR “Body Weights and Measures” OR “Body Measures” OR “Body Measure” OR “Body Size” OR “Body Weight” OR “Weight” OR “Waist Circumference” OR “Neck circumference” OR “Skinfold Thickness” OR “Waist-Hip Ratio” OR “Waist Hip Ratio” OR “DEXA” OR “Fetal Macrosomia” OR “Birth Weight”))	Publication date: “until 10/12/2024” Languages: “English” or “Spanish”

Table S2. Mesh terms used in the search strategy and their entry terms.

MeSH term	*Entry terms*
Pregnant women	Women, Pregnant; Pregnant Woman; Woman, Pregnant
Pregnancy	Pregnancies; Gestation
Mother-Child Relations	Mother Child Relations; Mother-Child Relation; Relation, Mother-Child; Relations, Mother-Child; Mother-Child Interaction; Interaction, Mother-Child; Interactions, Mother-Child; Mother Child Interaction; Mother-Child Interactions; Mother-Child Relationship; Mother Child Relationship; Mother-Child Relationships; Relationship, Mother-Child; Relationships, Mother-Child; Mother-Infant Interaction; Interaction, Mother-Infant; Interactions, Mother-Infant; Mother Infant Interaction; Mother-Infant Interactions; Mother-Infant Relations; Mother Infant Relations; Mother-Infant Relation; Relation, Mother-Infant; Relations, Mother-Infant
Exercise	Exercises; Physical Activity; Activities, Physical; Activity, Physical; Physical Activities; Exercise, Physical; Exercises, Physical; Physical Exercise; Physical Exercises; Acute Exercise; Acute Exercises; Exercise, Acute; Exercises, Acute; Exercise, Isometric; Exercises, Isometric; Isometric Exercises; Isometric Exercise; Exercise, Aerobic; Aerobic Exercise; Aerobic Exercises; Exercises, Aerobic; Exercise Training; Exercise Trainings; Training, Exercise; Trainings, Exercise
Infant	Infants
Child	Children
Child, Preschool	Preschool Child; Children, Preschool; Preschool Children
Physical Fitness	Fitness, Physical
Cardiorespiratory Fitness	Fitness, Cardiorespiratory
Physical Conditioning, Human	Conditioning, Human Physical; Human Physical Conditioning; Physical Training, Human; Human Physical Training; Training, Human Physical
Motor Skills	Motor Skill; Skill, Motor; Skills, Motor
Psychomotor Performance	Performance, Psychomotor; Performances, Psychomotor; Psychomotor Performances; Visual Motor Coordination; Coordination, Visual Motor; Coordination, Visual Motor; Motor Coordination, Visual; Motor Coordination, Visual; Visual Motor Coordination; Perceptual Motor Performance; Motor Performance, Perceptual; Motor Performances, Perceptual; Perceptual Motor Performances; Performance, Perceptual Motor; Performances, Perceptual Motor; Sensory Motor Performance; Motor Performance, Sensory; Motor Performances, Sensory; Performance, Sensory Motor; Performances, Sensory Motor; Sensory; Motor Performances
Muscle Strength	Strength, Muscle; Arthrogenic Muscle Inhibition; Arthrogenic Muscle Inhibitions; Inhibition, Arthrogenic Muscle; Muscle Inhibition, Arthrogenic
Muscle strength dynamometer	Dynamometer, Muscle Strength; Dynamometers, Muscle Strength; Muscle Strength Dynamometers
Range of motion, articular	Joint Range of Motion; Joint Flexibility; Flexibility, Joint; Range of Motion; Passive Range of Motion
Postural Balance	Musculoskeletal Equilibrium; Equilibrium, Musculoskeletal; Posture Balance; Balance, Posture; Posture Balances; Posture Equilibrium; Equilibrium, Posture; Posture Equilibriums; Postural Equilibrium; Equilibrium, Postural; Balance, Postural; Postural Control; Control, Postural; Postural Controls; Posture Control; Control, Posture; Posture Controls
Anthropometry	Body Composition; Body Weights and Measures
Body Composition	Body Compositions; Composition, Body; Compositions, Body
Body Constitution	Body Compositions; Composition, Body; Compositions, Body
Body Weights and Measures	Body Measures; Body Measure; Measure, Body; Measures, Body
Body Size	Body Sizes; Size, Body; Sizes, Body
Body Weight	Body Weights; Weight, Body; Weights, Body
Waist Circumference	Circumference, Waist; Circumferences, Waist; Waist Circumferences
Skinfold Thickness	Skinfold Thicknesses; Thickness, Skinfold; Thicknesses, Skinfold
Waist-Hip Ratio	Ratio, Waist-Hip; Ratios, Waist-Hip; Waist Hip Ratio; Waist-Hip Ratios; Waist-to-Hip Ratio; Ratio, Waist-to-Hip; Ratios, Waist-to-Hip; Waist to Hip Ratio; Waist-to-Hip Ratios
Birth Weight	Birth Weights; Weight, Birth; Weights, Birth; Birthweight; Birthweights

Table S3. Main characteristics of the studies included in the systematic review.

Study	Sample N	Intervention	Kind of exercise: AE	RE	CE	S/PF	Frequency, duration, intensity, and supervision	Moment of assessment	Outcomes: Body composition: Body size	Adiposity	Lean mass	Apgar Score	Motor fitness	Results (IG Vs CG)
Aktan 2021[1]	N: 64<br>IG1: 21<br>IG2: 21<br>CG: 22	Exercise:<br>IG1:<br>24 - 36 GW<br>IG2:<br>32 – 36 GW		X		X	IG1: F: 2 days/week (-)<br>D: 60 min<br>I: 12-14 RPE<br>IG2: F:1 day/week (-)<br>D: 60 min<br>I: -	Birth	X			X		↑ 1-min Apgar score in IG1 (p=0.013)
Avery 1997[2]	N: 29<br>IG: 15<br>CG: 14	Exercise: (-)	X				F: 3-4 days/week (twice supervised)<br>D: 30 min<br>I: 70%HRmax	Birth	X			X		No statistical differences
Baciuk 2008[3]	N: 70<br>IG:33<br>CG: 37	Exercise:<br>18/20 –<br>35/36 GW	X				F: 3 days/week (supervised)<br>D: 50 min<br>I: 70%HRmax	Birth	X			X		No statistical differences
Baena 2022[4]	N: 136<br>IG: 50<br>CG: 86	Exercise:<br>16/17 GW -<br>delivery			X		F: 3 days/week (supervised)<br>D: 60 min<br>I: 12-16 RPE	Birth	X			X		No statistical differences
Barakat 2009a[5]	N: 142<br>IG: 72<br>CG: 70	Exercise:<br>12/13 GW -<br>delivery		X			F: 3 days/week (supervised)<br>D: 35-40 min<br>I: <60%HRmax	Birth	X			X		No statistical differences
Barakat 2009b[6]	N: 142<br>IG: 72<br>CG: 70	Exercise:<br>12/13 GW -<br>delivery		X			F: 3 days/week (supervised)<br>D: 36 min<br>I: ≤ 80%HRmax	Birth	X			X		No statistical differences
Barakat 2011[7]	N: 67<br>IG: 34<br>CG: 33	Exercise: 6/9<br>– 38/39			X	X	F: 3 days/week (supervised)<br>D: 35-45 min<br>I: 70%HRmax	Birth	X			X		No statistical differences
Barakat 2012[8]	N: 83<br>IG: 40<br>CG: 43	Exercise: 6/9<br>– 38/39			X	X	F: 3 days/week (supervised)<br>D: 35-45 min<br>I: 70%HRmax	Birth	X			X		No statistical differences
Barakat 2013[9]	N: 428<br>IG: 210<br>CG: 218	Exercise:<br>10/12 –<br>38/39 GW			X		F: 3 days/week (supervised)<br>D: 50-55 min<br>I: 70%HRmax; 10-12 RPE	Birth	X			X		↓ Macrosomia (p=0.002)

Barakat 2014a[10]	N: 200<br>IG: 107<br>CG: 93	Exercise:<br>9/13 GW -<br>Delivery.	X			X	F: 3 days/week (supervised)<br>D: 55-60 min<br>I: 55-60% HRmax /12-13 RPE	Birth	X			X	No statistical differences
Barakat 2014b[11]	N: 290<br>IG: 138<br>CG: 152	Exercise:<br>8/10 – 38/39<br>GW			X	X	F: 3 days/week (supervised)<br>D: 55-60 min<br>I: 60-75%HRmax	Birth	X			X	No statistical differences
Barakat 2016[12]	N: 765<br>IG: 382<br>CG: 383	Exercise:<br>9/11 GW –<br>delivery			X	X	F: 3 days/week (supervised)<br>D: 50-55 min<br>I: 30-60%1RM	Birth	X			X	↓ Macrosomia (1.8% vs 4.7%; $p=0.03$)
Bisson 2015[13]	N: 48<br>IG: 24<br>CG: 24	Exercise: (-)			X		F: once/week (supervised) +<br>twice no supervised	Birth	X				No statistical differences
Brik 2019[14]	N: 85<br>IG: 42<br>CG: 43	Exercise:<br>9 GW –<br>delivery			X	X	F: 3 days/week (supervised)<br>D: 60 min<br>I:55-60%HRmax	Birth	X			X	No statistical differences
Cavalcante 2009[15]	N: 71<br>IG: 34<br>CG: 37	Exercise:<br>16/20 GW -<br>delivery	X				F: 3 days/week (supervised)<br>D: 50 min<br>I: 70%HRmax	Birth	X				No statistical differences
Chiavaroli 2018[16]	N: 61<br>IG: 38<br>CG: 23	Exercise:<br>20 GW -<br>delivery	X				F: 5 days/week (no supervised)<br>D: 40 min<br>I: 65%VO2max	1 year	X	X			No statistical differences
Claiborne 2024[17]	N: 358<br>IG1: 91<br>IG2: 59<br>IG3: 65<br>CG: 83	Exercise: 16<br>GW –<br>delivery	X	X	X	X	F: 3 days/week (supervised)<br>D: 50 min<br>IG1: I: 40%–59% VO_2peak,<br>12–15 RPE<br>IG2: 12–15 RPE, 60%1RM<br>IG3: %–59% VO_2peak, 12–15<br>RPE, 60%1RM	Birth	X	X		X	IG1: ↑ ponderal index (z-score) ($p<0.05$).<br>IG3: ↑ ponderal index (z-score) and weight to length ratio (z-score) (both $p<0.05$).
Clapp 2000[18]	N: 46<br>IG: 22<br>CG: 24	Exercise:<br>8/9GW -<br>delivery	X				F: 3-5 days/week (-)<br>D: 20 min<br>I: 55-60%VO_2max	Birth and 5 days	X	X	X		↑ Birth weight, length, and lean mass (all $p=0.05$)
Clapp 2002[19]	N: 75<br>IG1: 24<br>IG2: 25<br>IG3: 26	Exercise:<br>8 GW –<br>delivery	X				I: 55-65% VO_2max<br>IG1: F: 5 days/week (-)<br>D: 20-60 min<br>IG2: F: 5 days/week (-)<br>D: 40 min<br>IG3: F: 5 days/week (-)<br>D: 60-20 min	Birth	X	X		X	GI1 vs GI2 and GI3: ↓ Birth weight, adiposity, ponderal index, head/abdominal ratio (all $p<0.0001$) and length ($p<0.001$)<br>GI1 vs GI2 and GI3: ↑ Lean mass ($p<0.0001$)
Clark 2019[20]	N: 36<br>IG: 14	Exercise:	X				F: 3 days/week (supervised)<br>D: 60 min	Birth	X	X		X	↑ Head circumference ($p=0.02$)

	CG: 22	16 GW -<br>delivery					I: 12-14 RPE						
Collings<br>1983[21]	N: 20<br>IG: 12<br>CG: 8	Exercise:<br>7/19 - 37<br>GW	X			X	F: 3 days/week (supervised)<br>D: 50 min<br>I: 65-70%VO_2max	Birth	X			X	No statistical differences
Cordero<br>2015[22]	N: 257<br>IG: 101<br>CG: 156	Exercise:<br>10/14 GW -<br>delivery			X		F: 3 days/week (supervised)<br>D: 50-60 min<br>I: 60%HRmax	Birth	X				No statistical differences
Da Silva<br>2017[23]	N: 492<br>IG: 85<br>CG: 407	Exercise:<br>16/20 -<br>32/36 GW			X	X	F: 3 days/week (supervised)<br>D: 60 min<br>I: 12-14 RPE	Birth	X				No statistical differences
Daly<br>2017[24]	N: 87<br>IG: 44<br>CG: 43	Exercise: (-)			X		F: 3 days/week (supervised)<br>D: 50-60 min<br>I: (-)	Birth	X			X	No statistical differences
De Barros<br>2010[25]	N: 64<br>IG: 32<br>CG: 32	Exercise +<br>diet: 27 -<br>delivery		X			F: 3 days/week (supervised)<br>D: 30-40 min<br>I: RPE 0-10 (5-6)	Birth	X				No statistical differences
De Oliveria<br>2012[26]	N: 187<br>IG1:62<br>IG2: 63<br>CG: 62	Exercise:<br>13/20 GW –<br>delivery	X				F: 3 days/week (supervised)<br>D: >15 min<br>I:60-80%HRmax / 12-16 RPE	Birth	X				No statistical differences
Dias<br>2011[27]	N: 33<br>IG: 16<br>CG: 17	Exercise:<br>9 GW –<br>delivery				X	F: Twice/day (no supervised)<br>+1 day/week (supervised)<br>D: 30 min/week<br>I: -	Birth	X			X	No statistical differences
Facchinetti<br>2019[28]	N: 156<br>IG: 81<br>CG: 75	Exercise +<br>diet:<br>20 GW –<br>delivery	X				F: 4 days/week (no supervised)<br>D: 30 min<br>I: -	Birth	X			X	↓ Birth weight (p=0.002), LGA (2% vs 9%; p=0.02) and macrosomia (0 vs 8%; p=0.009)
Ferrari<br>2023[29]	N: 70<br>IG: 45<br>CG: 25	Exercise +<br>diet: 14->36<br>GW			X		F: 2 days/week (supervised)<br>D: 60-90 min<br>I: -	Birth	X	X	X	X	↑ Birth length (p=0.048)
Garnaes<br>2017[30]	N: 74<br>IG: 38<br>CG: 36	Exercise:<br>-			X	X	F: 3 days/week (+2 non supervised)<br>D: 60 min<br>I: 12-15 RPE	Birth	X	X		X	No statistical differences
Garshasbi<br>2005[31]	N: 212<br>IG: 107<br>CG: 105	Exercise: (-)	?	?	?	?	F: 3 days/week (supervised)<br>D: 60 min<br>I: HR<140bpm	Birth	X				No statistical differences

Haakstad 2014[32]	N: 74<br>IG: 21<br>CG: 53	Exercise:<br>24/26-36/38 GW			X		F: 2 days/week (supervised) + 1, 30min (no supervised)<br>D: 60 min<br>I: 12-14 RPE	Birth	X			X		↓ Birth length (p=0.04)<br>↑ 1-min Apgar (p=0.02)
Hall 1987[33]	N: 845<br>IG1: 82<br>IG2: 309<br>IG3: 61<br>CG: 393	Exercise:<br>? - delivery			X	X	F: 3 days/week (supervised)<br>D: 45 min<br>I: 85% HRmax	Birth	X			X		↑ 1 and 5-min Apgar score in IG3 vs IG1, IG2, CG (p<0.05)
Hellenes 2015[34]	N: 336<br>IG: 188<br>CG: 148	Exercise + diet:<br>20 - 36 GW			X	X	F: 1 day/week, 60min (supervised) + 2, 45min (no supervised)<br>D: 45-60 min<br>I: 55-60%HRmax	Birth and 18 months	X				X	↓ Fine motor skills in boys but not girls (95%CI: 10.3–11.0 vs 11.0–11.9; p=0.01)
Hopkins 2010[35]	N: 84<br>IG: 47<br>CG: 37	Exercise:<br>20 GW – delivery	X				F: 5 days/week (no supervised)<br>D: 40 min<br>I: 65% VO2max	Birth and 17 days	X	X	X			↓ BMI at birth (p<0.05)<br>↓ Weight and lean mass at 17 days (p<0.05)
Hui 2011[36]	N: 190<br>IG: 102<br>CG: 88	Exercise:<br>20/26 – 36 GW			X	X	F: 3-5 days/week (no supervised)<br>D: 20 min<br>I: (-)	Birth	X					No statistical differences
Huifen 2022[37]	N: 89<br>IG: 43<br>CG: 46	Exercise + diet: 31-37 GW		X			F: 3 days/week (supervised)<br>D: 50-60 min<br>I: 13-14 RPE	Birth	X			X		No statistical differences
Jevtovic 2023[38]	N: 41<br>IG1: 10<br>IG2: 11<br>IG3: 9<br>CG: 11	Exercise: 16 GW – delivery	X	X	X	X	F: 3 days/week (supervised)<br>D: 50 min<br>IG1: I: 12–15 RPE 40%–59% VO_2peak,<br>IG2: 12–15 RPE, 60%1RM<br>IG3: %–59% VO_2peak, 12–15 RPE, 60%1RM	Birth	X	X		X		No statistical differences
Jevtovic 2024a[39]	N: 13<br>IG: 7<br>CG: 6	Exercise: 16 GW – delivery	X	X	X	X	F: 3 days/week (supervised)<br>D: 50 min<br>IG: I: 12–15 RPE 40%–59% VO_2peak, 60%1RM	Birth	X	X		X		No statistical differences
Jevtovic 2024b[40]	N: 41<br>IG: 30<br>CG: 11	Exercise: 16 GW – delivery	X	X	X	X	F: 3 days/week (supervised)<br>D: 50 min<br>IG: I: 12–15 RPE 40%–59% VO_2peak, 60%1RM	Birth	X	X		X		No statistical differences

Jin 2022[41]	N: 131<br>IG: 65<br>CG: 66	Exercise + diet: -				X	F: 10 times/week (-)<br>D: 15 min<br>I: 50-70%HRmax	Birth	X					No statistical differences
Jovanovic 1989[42]	N: 19:<br>IG: 10<br>CG: 9	Exercise + diet: (-)	X				F: 3 days/week (supervised)<br>D: 20 min<br>I: 70%HRmax (<140 bpm)	Birth	X					No statistical differences
Kihlstrand 1999[43]	N: 242<br>IG: 122<br>CG: 120	Exercise: 19 GW – delivery				X	F: once/week (supervised)<br>D: 60 min<br>I:	Birth	X					No statistical differences
Marquez 2000[44]	N: 15<br>IG: 9<br>CG: 6	Exercise: for 15 weeks			X		F: 3 days/week (supervised)<br>D: 60 min<br>I: < 156 bpm	Birth	X			X		No statistical differences
McDonald 2021[45]	N: 56<br>IG: 24<br>CG: 32	Exercise: 7/19 - 37 GW	X				F: 3 day/week (supervised)<br>D: 60 min<br>I: 40-59%VO_2peak	Birth and 1 month	X	X	X			↓ Adiposity (95%CI: 4.0, 10.5 vs 4.0, 9.5; p=0.02)
McDonald 2022[46]	N: 192<br>IG1: 62<br>IG2: 32<br>IG3: 37<br>CG: 61	Exercise: 13/16 - delivery	X	X	X		F: 3 days/week (supervised)<br>D: 60 min<br>I: 40–59% VO2peak; 15 RPE	Birth	X			X		Maternal exercise predicts birth weight (p=0.03)
McMillan 2019[47]	N: 60<br>IG: 33<br>CG: 27	Exercise: -	X				F: 3 days/week (supervised)<br>D: 30 min<br>I: 40% HRR	1 month	X				X	↑ Locomotion skills (p=0.02)
Menichini 2020[48]	N: 82<br>IG: 36<br>CG: 46	Exercise + diet: -	X				F: 4 days/week (no supervised)<br>D: 30 min<br>I: -	Birth	X			X		↓ LGA (8.3% vs 26.1%; p=0.04)
Mortaji 2023[49]	N: 74<br>IG: 42<br>CG:32	Exercise + diet: 12 GW – delivery	X				F: 3-7 days/week (1 supervised)<br>D: 25-40 min<br>I: -	Birth and 1 year	X				X	No statistical differences
Murtezani 2014[50]	N: 63<br>IG: 30<br>CG: 33	Exercise: 20 GW - delivery			X		F: 3 days/week (supervised)<br>D: 40-45 min<br>I: 12-14 RPE	Birth	X			X		↑ 1-min Apgar score (p=0.036) and 5-min Apgar score (p=0.015)
Nascimento 2011[51]	N: 80<br>IG: 39<br>GC: 41	Exercise: 9 – 36 GW		X		X	F: 5 days/week (supervised)<br>D: 40 min<br>I: light/moderate	Birth	X					No statistical differences
Oostdam 2012[52]	N: 121<br>IG: 62<br>CG: 59	Exercise: -			X		F: 2 days/week (supervised)<br>D: 60 min<br>I: 30-60% 1RM	Birth	X					No statistical differences

Perales 2014[53]	N: 166<br>IG: 83<br>CG: 83	Exercise:<br>9/11 GW -<br>delivery		X	X	F: 3 days/week (supervised)<br>D: 55-60 min<br>I: 55-60%HRmax	Birth	X		X	No statistical differences
Price 2012[54]	N: 62<br>IG: 31<br>CG: 31	Exercise:<br>12/14 – 36<br>GW/delivery	X			F: 4 days/week (supervised)<br>D: 45-60 min<br>I: 12-14 RPE	Birth	X		X	No statistical differences
Raper 2021[55]	N: 97<br>IG: 30<br>GC: 67	Exercise:<br>13/16 GW –<br>delivery		X		F: 3 days/week (supervised)<br>D: 50 min<br>I: 40-59%VO_2peak<br>60-80%VO_2max<br>12-14 RPE	Birth	X	X		No statistical differences
Rodriguez 2017[56]	N: 129<br>IG: 65<br>CG: 64	Exercise:<br>20 - 37 GW		X		F: 3 days/week (supervised)<br>D: 60 min<br>I: 12-14 RPE	Birth	X			↓ Birth weight (p=0.011)
Rodriguez 2019[57]	N: 129<br>IG: 65<br>CG: 64	Exercise:<br>20 - 37 GW		X		F: 3 days/week (supervised)<br>D: 60 min<br>I: 12-14 RPE	Birth	X			↓ Birth weight (p=0.011)
Roland 2023[58]	N: 132<br>IG: 87<br>CG: 45	Exercise: 15<br>GW -<br>delivery		X		F: 3 days/week (supervised)<br>D: 60 min<br>I: moderate	Birth	X		X	No statistical differences
Ruchat 2012[59]	N: 94<br>IG1: 23<br>IG2: 26<br>CG: 45	Exercise +<br>diet: 16/20<br>GW -<br>delivery	X			F: 3-4 days/week (supervised)<br>D: 25-40 min<br>I: 30%HRmax (IG1)<br>70%HRmax (IG2)	Birth	X			No statistical differences
Ruiz et al. 2013[60]	N: 962<br>IG: 481<br>CG: 481	Exercise:<br>9 – 38/39<br>GW		X		F: 3 days/week (supervised)<br>D: 50-55 min<br>I: 60%HRmax	Birth	X		X	↓ Macrosomia (p=0.007)
Seneviratne 2016[61]	N: 80<br>IG: 40<br>CG: 40	Exercise +<br>diet:<br>8/9 – 37 GW	X			F: 3-5 days/week (no supervised)<br>D: 15-30 min<br>I: 40-59%VO_2R	Birth	X		X	No statistical differences
Sklempe 2018[62]	N: 38<br>IG: 18<br>CG: 20	Exercise:<br>20/30 – 38<br>GW		X	X	F: twice/week (supervised)<br>D: 50-55 min<br>I: 65-75% HRmax	Birth	X		X	↑ BMI at birth (p=0.035)
Stafne 2012[63]	N: 855<br>IG: 429<br>CG: 426	Exercise: 20<br>– 36 GW		X	X	F: once/week (supervised) +<br>45 min twice/week (no supervised)<br>D: 60 min<br>I: 13-14 RPE	Birth	X		X	No statistical differences

Strom 2022[64]	N: 35<br>IG: 19<br>CG: 10	Exercise: 16GW - delivery	X			F: 3 days/week (supervised)<br>D: 60 min<br>I: 30%VO_2peak	1 month	X	X	X		↓ triceps and subscapular skinfolds (p=0.008 and p= 0.04, respectively), sum of skinfolds (p=0.01) and % body fat (p=0.006)
Tomić 2013[65]	N: 334<br>IG: 166<br>CG: 168	Exercise: 6/8 GW - delivery	X			F: 3 days/week (supervised)<br>D: 50 min<br>I: 60-75%HRmax	Birth	X				↓ Macrosomia (6% vs 12.5%; p=0.048)
Wang 2017[66]	N: 226<br>IG: 112<br>CG: 114	Exercise: 12/18 - 37 GW	X			F: 3 days/week (supervised)<br>D: 30 min<br>I: 55-65% HRmax<br>12-14 RPE	Birth	X			X	↓ Birth weight (p=0.049)
Yang 2023[67]	N: 355<br>IG: 178<br>CG: 177	Exercise + diet: 16 - >34 GW	X		X	F: 3-5 days/week (no supervised)<br>D: 30-60min<br>I: low-moderate	Birth	X				↓ Birth weight and length (both p<0.01)
Yang 2024[68]	N: 308<br>IG: 161<br>CG: 147	Exercise + diet: 16 - >34 GW	X		X	F: 3-5 days/week (no supervised)<br>D: 30-60min<br>I: low-moderate	Birth	X				↓ Macrosomia and large for gestational age
Yekefallah 2021[69]	N: 70<br>IG: 35<br>CG: 35	Exercise: 26/28 - 37 GW		X	X	F: 2 days/week (supervised)<br>D: 75 min<br>I: 60-75%HRmax	Birth	X			X	↓ Birth weight (p=0.001)<br>↑ 1-min Apgar score (p=0.01) and 5-min Apgar score (p<0.0001)

*Abbreviations: 1RM: one repetition maximum; 95%CI: confidence interval at 95%; AE: aerobic exercise; BMI: body mass index; CE: concurrent exercise; CG: control group; D: duration; DM2: diabetes mellitus 2; F: frequency; GD: gestational diabetes; GW: gestational week; HR: heart rate; HRmax: maximum heart rate; HRR: heart rate reserve; I: intensity; IG: intervention group; LGA: large for gestational age: Min: minute/s; OR: odds ratio; PF: pelvic floor exercise; RE: resistance exercise; RPE: Rate of Perceived Exertion; S: stretching; VO_2max: maximal oxygen consumption; VO_2peak: oxygen consumption peak; VO_2R: oxygen consumption reserve.

Table S4. Influence on neonatal outcomes by maternal age and pre-pregnancy body mass index.

	Maternal Age						Pre-pregnancy body mass index								
	≤ 30 years			> 30 years			< 25 kg/m²			25-29.9 kg/m²			≥ 30 kg/m²		
	S (EG)	ES (p value)	I^2 (%)	S (EG)	ES (p value)	I^2 (%)	S (EG)	ES (p value)	I^2 (%)	S (EG)	ES (p value)	I^2 (%)	S (EG)	ES (p value)	I^2 (%)
**Apgar score at 1 minute**															
Overall	7(8)	**0.18(0.03)**	0	14(16)	**0.09(0.03)**	0	15	**0.09(0.01)**	0	5(8)	0.09(0.17)	0	2	0.35(0.23)	64
AE	3	0.10(0.59)	0	3	0.08(0.33)	0	2	0.03(0.84)	0	4	0.10(0.24)	0	0	NA	NA
RE	4(5)	0.23(0.06)	30	2	0.09(0.48)	0	4	0.19(0.05)	2	2(3)	0.17(0.39)	46	0	NA	NA
CE	0	NA	NA	11	**0.09(0.03)**	0	9	0.08(0.06)	0	1	0.00 (1.00)	NA	2	0.35(0.23)	64
**Apgar score at 5 minutes**															
Overall	8(9)	0.31(0.08)	71	14	0.03(0.45)	26	16	0.09(0.20)	*60*	5(8)	0.09(0.21)	8	2	0.47(0.08)	56
AE	3	**0.39(0.04)**	0	3	0.07(0.38)	0	2	0.03(0.84)	8	4	0.17(0.12)	29	0	NA	NA
RE	4(5)	0.24(0.34)	83	1	0.09(0.51)	0	4	0.35(0.17)	85	2(3)	-0.02(0.88)	0	0	NA	NA
CE	1	0.77(0.16)	NA	9	0.02(0.69)	41	10	-0.01(0.84)	*15*	1	0.00 (1.00)	NA	2	0.47(0.08)	56
**Body birth weight**															
Overall	15(19)	-0.05(0.59)	61	33(36)	**-0.10(0.03)**	64	27(29)	**-0.09(0.04)**	47	14(19)	-0.04(0.64)	40	7	-0.22(0.30)	89
AE	7(8)	-0.11(0.12)	0	11(12)	**-0.28(0.02)**	77	8(10)	**-0.17(0.006)**	0	8	-0.14(0.10)	6	2	-0.75(0.22)	97
RE	5(6)	0.01(0.96)	86	5	0.01(0.91)	0	4	0.04(0.89)	85	4(5)	-0.05(0.86)	73	1	0.06(0.79)	NA
CE	5	-0.01(0.87)	0	19	-0.02(0.62)	38	15	-0.05(0.27)	44	6	0.03(0.64)	0	4	0.01(0.91)	28
**Body birth length**															
Overall	6(8)	-0.07(0.84)	94	12(13)	0.12(0.33)	83	11(12)	-0.17(0.35)	93	5(7)	0.13(0.14)	0	2	-0.21(0.23)	0
AE	4	-0.19(0.77)	94	3(4)	-0.01(0.94)	0	4(5)	-0.22(0.62)	96	3	0.02(0.92)	0	0	NA	NA
RE	2	-0.03(0.88)	10	2	1.79(0.40)	98	2	-0.18(0.17)	0	1	0.38(0.38)	NA	0	NA	NA
CE	2	0.08(0.47)	0	7	0.04(0.69)	47	5	0.02(0.85)	42	3	0.19(0.18)	21	2	-0.21(0.23)	0
**Head circumference at birth**															
Overall	2	0.37(0.39)	81	6	**-0.11(0.04)**	0	4	-0.11(0.06)	0	3	0.14(0.54)	68	2	-0.01(0.93)	0
AE	1	**0.87(0.02)**	NA	2	-0.22(0.07)	0	1	-0.24(0.09)	NA	2	0.32(0.54)	83	0	NA	NA
CE	1	0.00(1.00)	NA	4	-0.09(0.16)	0	3	-0.08(0.19)	0	1	0.00(1.00)	NA	2	-0.01(0.93)	0

*Abbreviations: AE: aerobic training; CE = concurrent training; ES: effect size; NA: not applicable; RE: resistance training; S (EG): number of studies (experimental groups).
*Judgement: normal weight: BMI < 25 kg/m²; overweight: BMI 25-29.9 kg/m²; obese ≥ 30 kg/m²

Table S5. Publication Bias Assessment

	Apgar 1'		Apgar 5'		Body birth weight		Body birth length		Head Circumference	
	value	p	value	p	value	p	value	p	value	p
**Egger's Regression**	1.584	0.113	2.640	0.008	-0.118	0.906	3.008	0.003	0.981	0.327

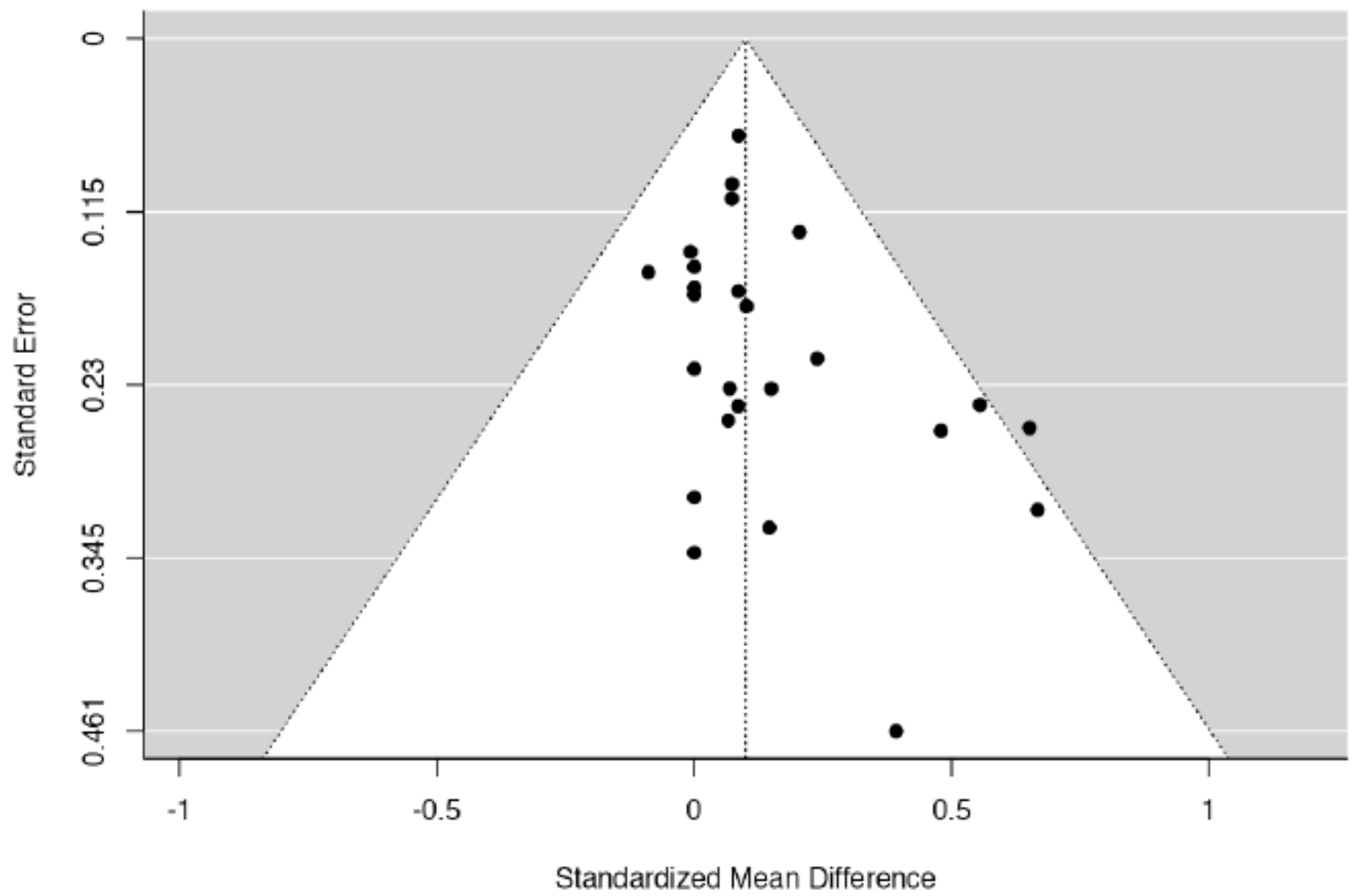

Figure S1.1. Funnel-plot of Apgar score at 1 minute.

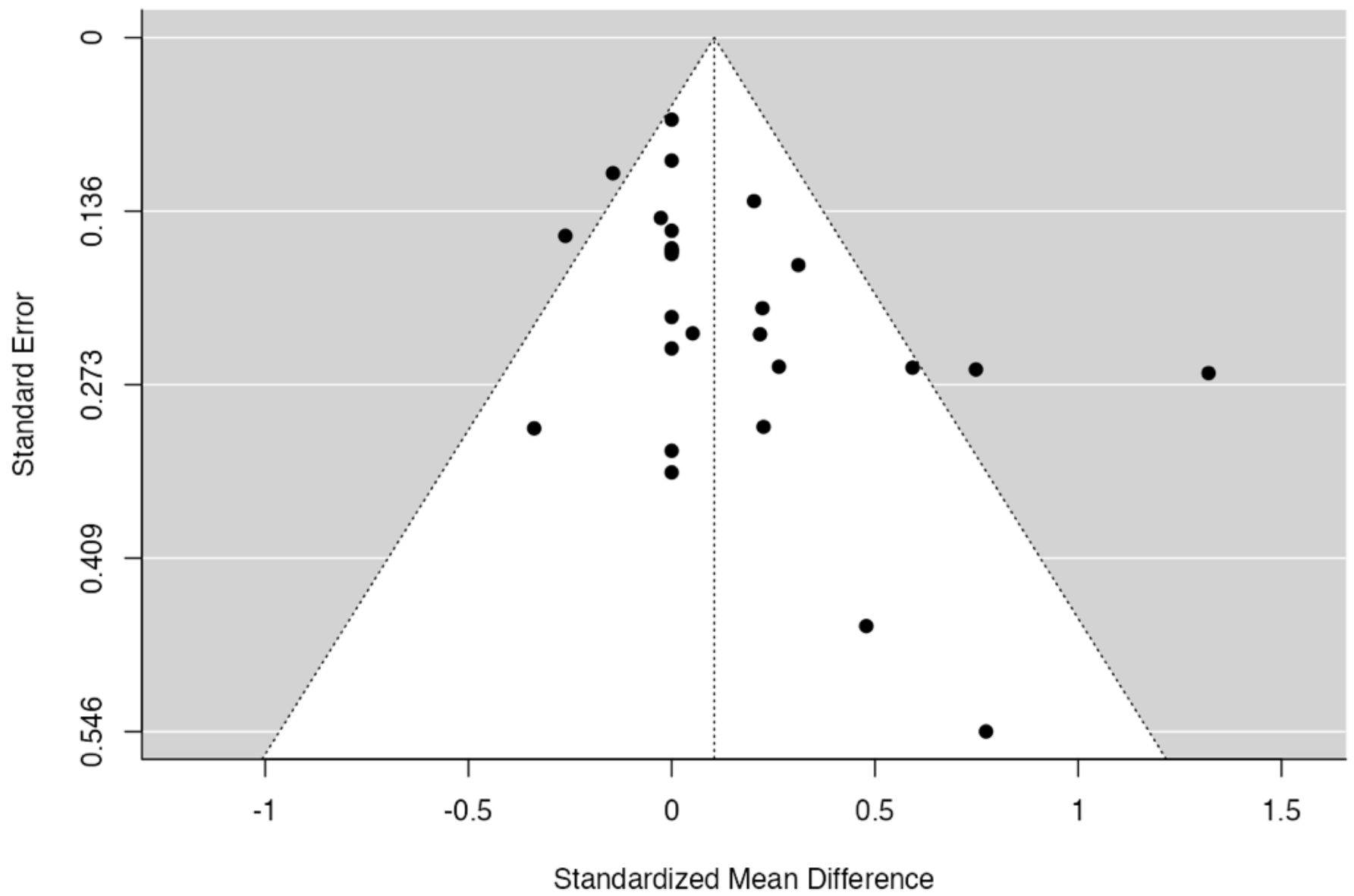

Figure S1.2. Funnel-plot of Apgar score at 5 minutes.

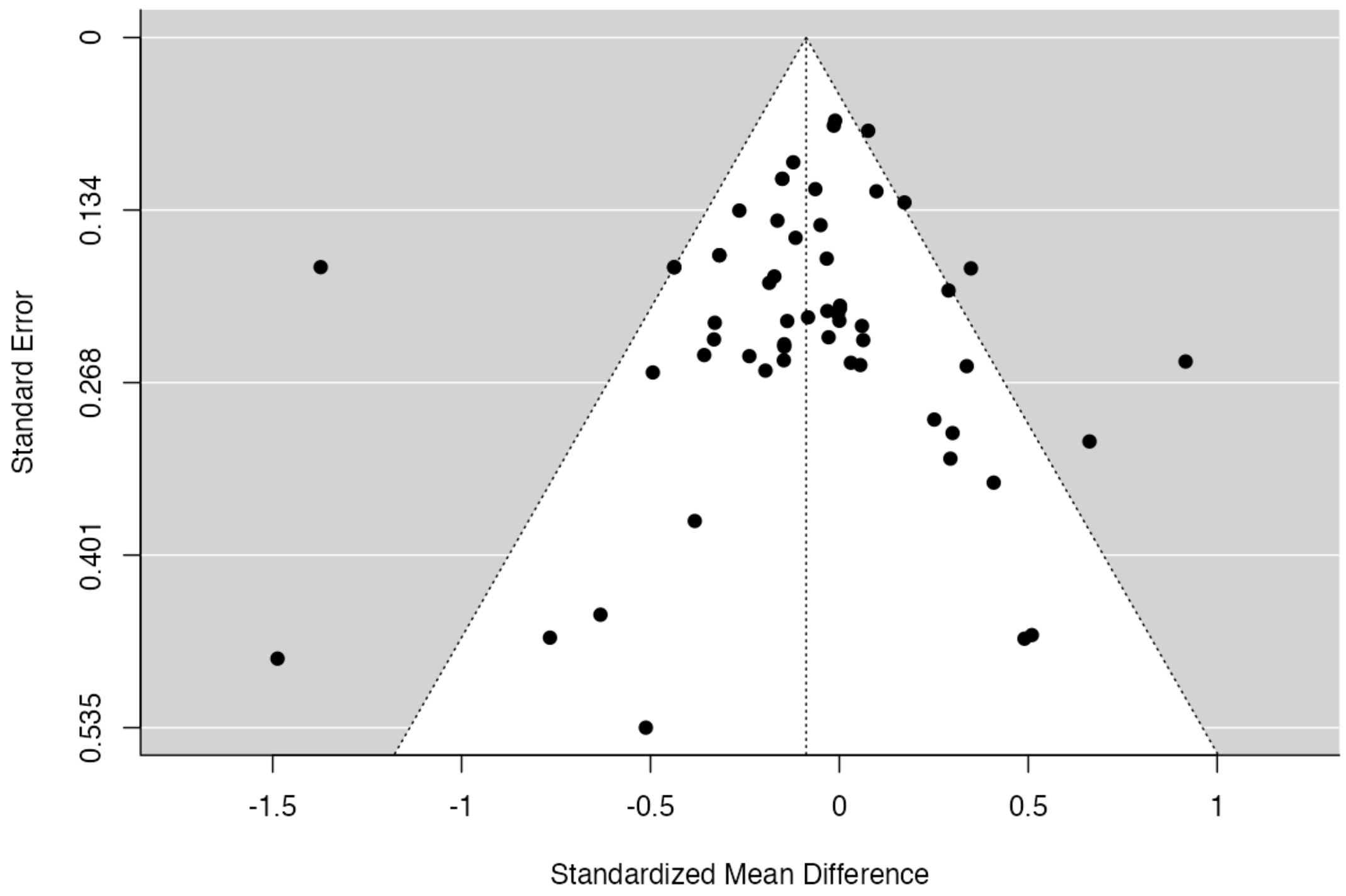

Figure S1.3. Funnel-plot of body birth weight.

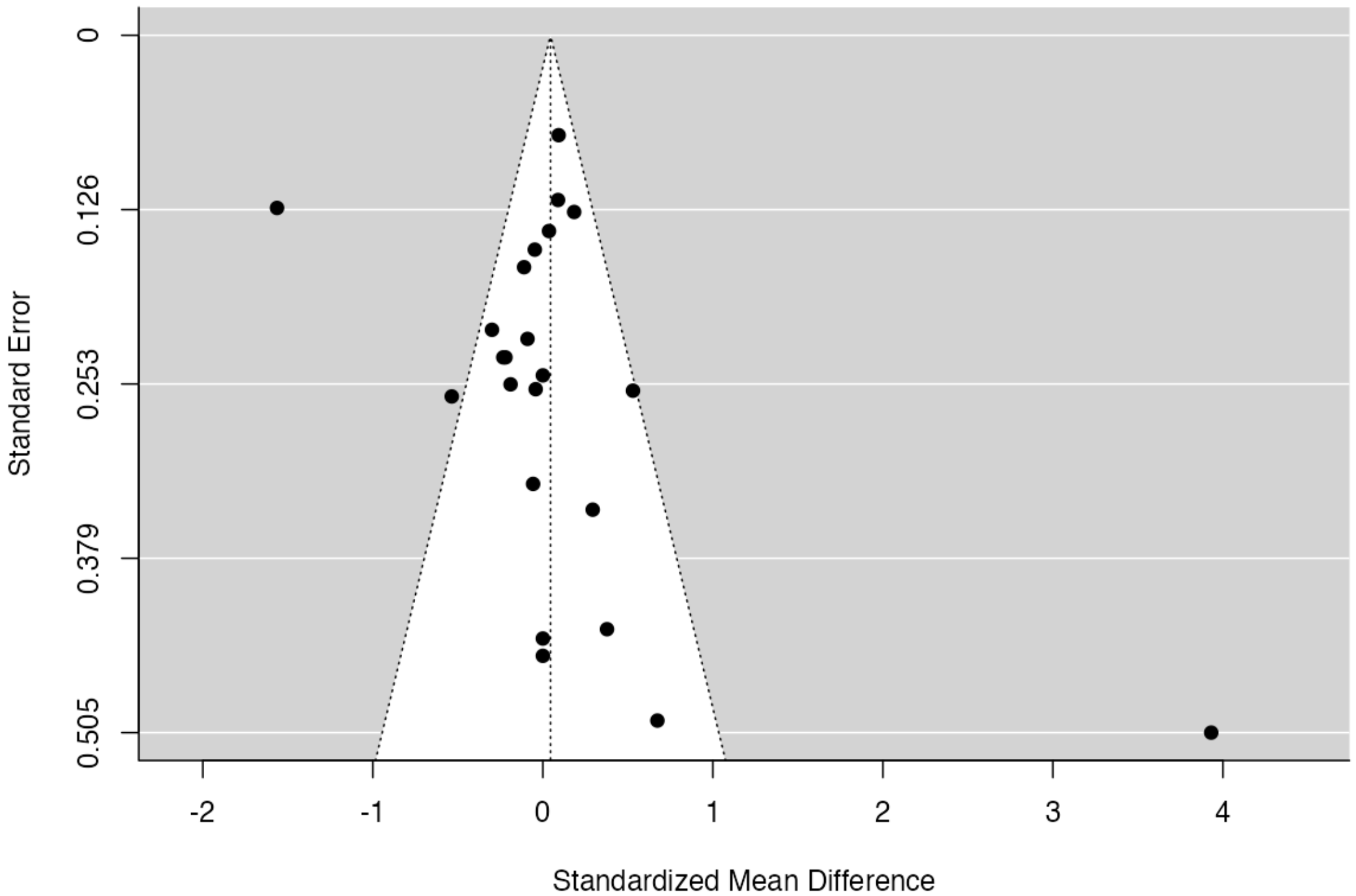

Figure S1.4. Funnel-plot of body birth length.

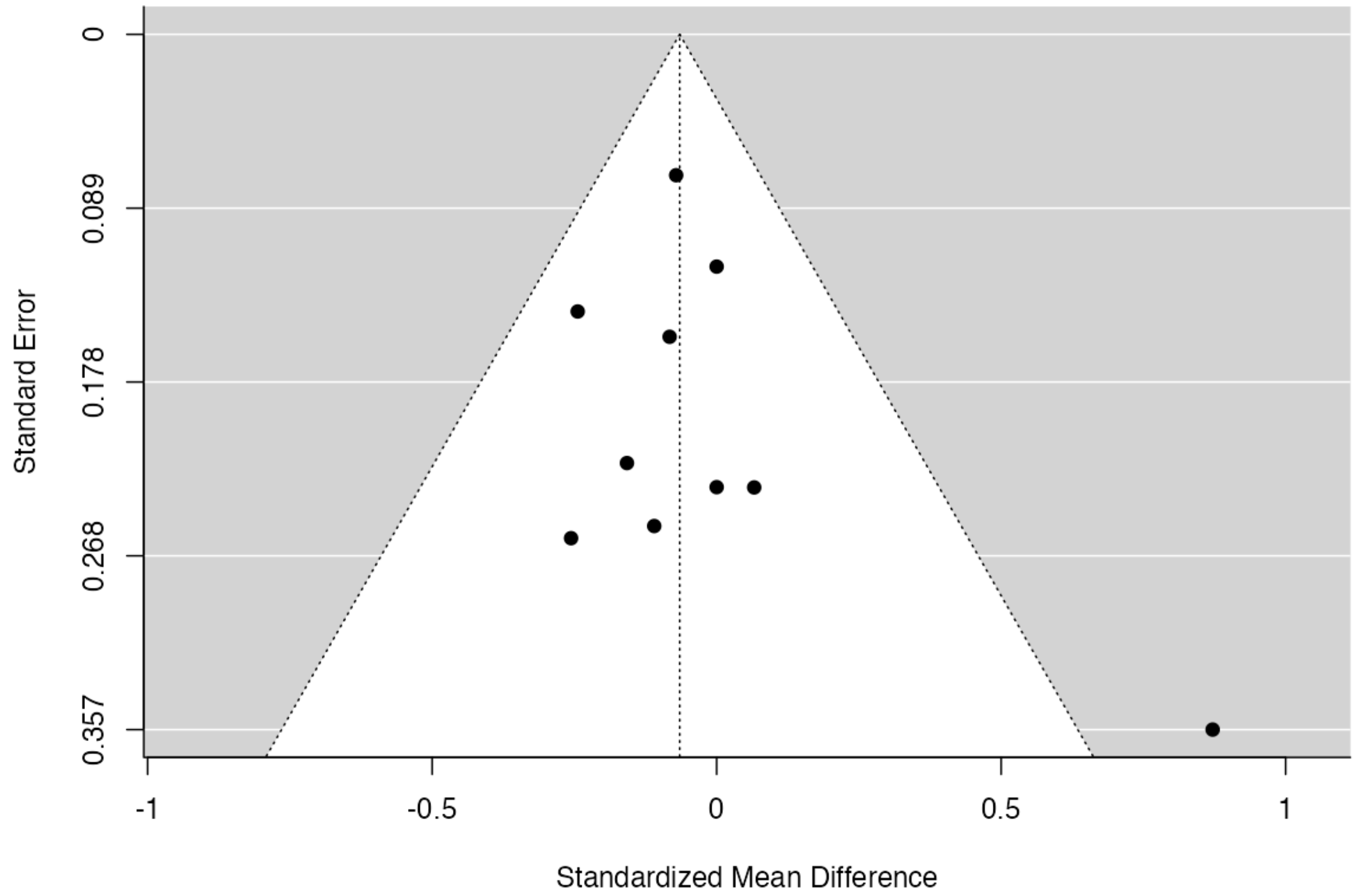

Figure S1.5. Funnel-plot of head circumference at birth.

Figure S1. Funnel-plot of neonatal outcomes. These funnel plots depict the standardized mean difference of trials versus their standard error.

1. Aktan B, Kayıkçıoğlu F, Akbayrak T. The comparison of the effects of clinical Pilates exercises with and without childbirth training on pregnancy and birth results. *Int J Clin Pract*. 2021;75(10):e14516. doi:10.1111/ijcp.14516

2. Avery MD, Leon AS, Kopher RA. Effects of a partially home-based exercise program for women with gestational diabetes. *OBSTETRICS AND GYNECOLOGY*. 1997;89(1):10-15. doi:10.1016/S0029-7844(97)84256-1

3. Baciuk EP, Pereira RI, Cecatti JG, Braga AF, Cavalcante SR. Water aerobics in pregnancy: cardiovascular response, labor and neonatal outcomes. *Reprod Health*. 2008;5(1):10. doi:10.1186/1742-4755-5-10

4. Baena-García L, de la Flor-Alemany M, Coll-Risco I, Reoyo OR, Aranda P, Aparicio VA. A concurrent prenatal exercise program increases neonatal and placental weight and shortens labor: The GESTAFIT project. *Scand J Med Sci Sports*. 2023;33(4):465-474. doi:10.1111/sms.14298

5. Barakat R, Lucia A, Ruiz JR. Resistance exercise training during pregnancy and newborn's birth size: a randomised controlled trial. *Int J Obes (Lond)*. 2009;33(9):1048-1057. doi:10.1038/ijo.2009.150

6. Barakat R, Ruiz JR, Stirling JR, Zakynthinaki M, Lucia A. Type of delivery is not affected by light resistance and toning exercise training during pregnancy: a randomized controlled trial. *Am J Obstet Gynecol*. 2009;201(6):590.e1-590.e6. doi:10.1016/j.ajog.2009.06.004

7. Barakat R, Pelaez M, Montejo R, Luaces M, Zakynthinaki M. Exercise during pregnancy improves maternal health perception: A randomized controlled trial. *Am J Obstet Gynecol*. 2011;204(5):402.e1-402.e7. doi:10.1016/j.ajog.2011.01.043

8. Barakat R, Cordero Y, Coteron J, Luaces M, Montejo R. Exercise during pregnancy improves maternal glucose screen at 24–28 weeks: a randomised controlled trial. *Br J Sports Med*. 2012;46(9):656-661. doi:10.1136/bjsports-2011-090009

9. Barakat R, Pelaez M, Lopez C, Lucia A, Ruiz JR. Exercise during pregnancy and gestational diabetes-related adverse effects: a randomised controlled trial. *Br J Sports Med*. 2013;47(10):630-636. doi:10.1136/bjsports-2012-091788

10. Barakat, Perales M, Bacchi M, et al. A Program of Exercise Throughout Pregnancy. Is It Safe to Mother and Newborn? *Am J Health Promot*. 2014;29(1):2-8. doi:10.4278/ajhp.130131-QUAN-56

11. Barakat R, Pelaez M, Montejo R, Refoyo I, Coteron J. Exercise Throughout Pregnancy Does not Cause Preterm Delivery: A Randomized, Controlled Trial. *J Phys Act Health*. 2014;11(5):1012-1017. doi:10.1123/jpah.2012-0344

12. Barakat, Pelaez M, Cordero Y, et al. Exercise during pregnancy protects against hypertension and macrosomia: randomized clinical trial. *Am J Obstet Gynecol*. 2016;214(5):649.e1-8. doi:10.1016/j.ajog.2015.11.039

13. Bisson M, Alméras N, Dufresne SS, et al. A 12-Week Exercise Program for Pregnant Women with Obesity to Improve Physical Activity Levels: An Open Randomised Preliminary Study. Allison DB, ed. *PLoS One*. 2015;10(9):e0137742. doi:10.1371/journal.pone.0137742

14. Brik M, Fernández-Buhigas I, Martin-Arias A, Vargas-Terrones M, Barakat R, Santacruz B. Does exercise during pregnancy impact on maternal weight gain and fetal cardiac function? A randomized controlled trial. *Ultrasound Obstet Gynecol*. 2019;53(5):583-589. doi:10.1002/uog.20147

15. Cavalcante SR, Cecatti JG, Pereira RI, Baciuk EP, Bernardo AL, Silveira C. Water aerobics II: maternal body composition and perinatal outcomes after a program for low risk pregnant women. *Reprod Health*. 2009;6(1):1. doi:10.1186/1742-4755-6-1

16. Chiavaroli V, Hopkins SA, Derraik JGB, et al. Exercise in pregnancy: 1-year and 7-year follow-ups of mothers and offspring after a randomized controlled trial. *Sci Rep*. 2018;8(1):12915. doi:10.1038/s41598-018-30925-5

17. Claiborne A, Wisseman B, Kern K, et al. Exercise FITT-V during pregnancy: Association with birth outcomes. *Birth Defects Res*. 2024;116(4). doi:10.1002/bdr2.2340

18. Clapp JF, Kim H, Burciu B, Lopez B. Beginning regular exercise in early pregnancy: Effect on fetoplacental growth. *Am J Obstet Gynecol*. 2000;183(6):1484-1488. doi:10.1067/mob.2000.107096

19. Clapp JF, Kim H, Burciu B, Schmidt S, Petry K, Lopez B. Continuing regular exercise during pregnancy: Effect of exercise volume on fetoplacental growth. *Am J Obstet Gynecol*. 2002;186(1):142-147. doi:10.1067/mob.2002.119109

20. Clark E, Isler C, Strickland D, et al. Influence of aerobic exercise on maternal lipid levels and offspring morphometrics. *Int J Obes (Lond)*. 2019;43(3):594-602. doi:10.1038/s41366-018-0258-z

21. Collings CA, Curet LB, Mullin JP. Maternal and fetal responses to a maternal aerobic exercise program. *Am J Obstet Gynecol*. 1983;145(6):702-707. doi:10.1016/0002-9378(83)90576-8

22. Cordero Y, Mottola MF, Vargas J, Blanco M, Barakat R. Exercise Is Associated with a Reduction in Gestational Diabetes Mellitus. *Med Sci Sports Exerc*. 2015;47(7):1328-1333. doi:10.1249/MSS.0000000000000547

23. da Silva SG, Hallal PC, Domingues MR, et al. A randomized controlled trial of exercise during pregnancy on maternal and neonatal outcomes: results from the PAMELA study. *International Journal of Behavioral Nutrition and Physical Activity*. 2017;14(1):175. doi:10.1186/s12966-017-0632-6

24. Daly N, Farren M, McKeating A, O'Kelly R, Stapleton M, Turner MJ. A Medically Supervised Pregnancy Exercise Intervention in Obese Women. *Obstetrics & Gynecology*. 2017;130(5):1001-1010. doi:10.1097/AOG.0000000000002267

25. de Barros MC, Lopes MAB, Francisco RPV, Sapienza AD, Zugaib M. Resistance exercise and glycemic control in women with gestational diabetes mellitus. *Am J Obstet Gynecol*. 2010;203(6):556.e1-556.e6. doi:10.1016/j.ajog.2010.07.015

26. de Oliveria AS, Silva JLP, Tavares JS, Barros VO, Leite DFB, Amorim MMR. Effect of a physical exercise program during pregnancy on uteroplacental and fetal blood flow and fetal growth: a randomized controlled trial. *Obstet Gynecol*. 2012;120(2 Pt 1):302-310. doi:10.1097/AOG.0b013e31825de592

27. Dias LAR, Driusso P, Alta DLCC, Quintana SM, Bo K, Ferreira CHJ. Effect of pelvic floor muscle training on labour and newborn outcomes: a randomized controlled trial. *Braz J Phys Ther*. 2011;15(6):487-493. doi:10.1590/S1413-35552011005000011

28. Facchinetti F, Vijai V, Petrella E, et al. Food glycemic index changes in overweight/obese pregnant women enrolled in a lifestyle program: a randomized controlled trial. *Am J Obstet Gynecol MFM*. 2019;1(3):1-9. doi:10.1016/j.ajogmf.2019.100030

29. Ferrari N, Schmidt N, Schmidt L, et al. Effect of Lifestyle Interventions during Pregnancy on Maternal Leptin, Resistin and Offspring Weight at Birth and One Year of Life. *Biomedicines*. 2023;11(2). doi:10.3390/biomedicines11020447

30. Garnaes KK, Nyrnes SA, Salvesen KA, Salvesen O, Morkved S, Moholdt T. Effect of supervised exercise training during pregnancy on neonatal and maternal outcomes among overweight and obese women. Secondary analyses of the ETIP trial: A randomised controlled trial. *PLoS One*. 2017;12(3). doi:10.1371/journal.pone.0173937

31. Garshasbi A, Faghih Zadeh S. The effect of exercise on the intensity of low back pain in pregnant women. *International Journal of Gynecology & Obstetrics*. 2005;88(3):271-275. doi:10.1016/j.ijgo.2004.12.001

32. Haakstad LAH, Bo K. Exercise in pregnant women and birth weight: a randomized controlled trial. *BMC Pregnancy Childbirth*. 2011;11. doi:10.1186/1471-2393-11-66

33. Hall DC, Kaufmann DA. Effects of aerobic and strength conditioning on pregnancy outcomes. *Am J Obstet Gynecol*. 1987;157(5):1199-1203. doi:10.1016/S0002-9378(87)80294-6

34. Hellenes OM, Vik T, Løhaugen GC, et al. Regular moderate exercise during pregnancy does not have an adverse effect on the neurodevelopment of the child. *Acta Paediatr*. 2015;104(3):285-291. doi:10.1111/apa.12890

35. Hopkins SA, Baldi JC, Cutfield WS, McCowan L, Hofman PL. Exercise training in pregnancy reduces offspring size without changes in maternal insulin sensitivity. *J Clin Endocrinol Metab*. 2010;95(5):2080-2088. doi:10.1210/jc.2009-2255

36. Hui A, Back L, Ludwig S, et al. Lifestyle intervention on diet and exercise reduced excessive gestational weight gain in pregnant women under a randomised

controlled trial. Published online 2011:70-77. doi:10.1111/j.1471-0528.2011.03184.x

37. Huifen Z, Yaping X, Meijing Z, et al. Effects of moderate-intensity resistance exercise on blood glucose and pregnancy outcome in patients with gestational diabetes mellitus: A randomized controlled trial. *J Diabetes Complications*. 2022;36(5):108186. doi:10.1016/j.jdiacomp.2022.108186

38. Jevtovic F, Zheng D, Houmard JA, et al. Effects of Maternal Exercise Modes on Glucose and Lipid Metabolism in Offspring Stem Cells. *JOURNAL OF CLINICAL ENDOCRINOLOGY & METABOLISM*. 2023;108(7):E360-E370. doi:10.1210/clinem/dgad059

39. Jevtovic F, Collier DN, DeVente J, et al. Maternal exercise increases infant resting energy expenditure: preliminary results. *Int J Obes*. 2024;48(9):1347-1350. doi:10.1038/s41366-024-01560-0

40. Jevtovic F, Zheng D, Claiborne A, et al. Effects of maternal exercise on infant mesenchymal stem cell mitochondrial function, insulin action, and body composition in infancy. *Physiol Rep*. 2024;12(9). doi:10.14814/phy2.16028

41. Jin Y, Chen Z, Li J, Zhang W, Feng S. Effects of the original Gymnastics for Pregnant Women program on glycaemic control and delivery outcomes in women with gestational diabetes mellitus: A randomized controlled trial. *Int J Nurs Stud*. 2022;132. doi:10.1016/j.ijnurstu.2022.104271

42. Jovanovic-Peterson L, Durak EP, Peterson CM. Randomized trial of diet versus diet plus cardiovascular conditioning on glucose levels in gestational diabetes. *Am J Obstet Gynecol*. 1989;161(2):415-419. doi:10.1016/0002-9378(89)90534-6

43. Kihlstrand M, Stenman B, Nilsson S, Axelsson O. Water-gymnastics reduced the intensity of back/low back pain in pregnant women. *Acta Obstet Gynecol Scand*. 1999;78(3):180-185.

44. Marquez-Sterling S, Perry AC, Kaplan TA, Halberstein RA, Signorile JF. Physical and psychological changes with vigorous exercise in sedentary primigravidae. *Med Sci Sports Exerc*. 2000;32(1):58-62. doi:10.1097/00005768-200001000-00010

45. McDonald SM, Isler C, Haven K, et al. Moderate intensity aerobic exercise during pregnancy and 1-month infant Morphometry. *Birth Defects Res*. 2021;113(3):238-247. doi:10.1002/bdr2.1671

46. McDonald SM, Mouro S, Wisseman B, et al. Influence of prenatal exercise on the relationship between maternal overweight and obesity and select delivery outcomes. *Sci Rep*. 2022;12(1). doi:10.1038/s41598-022-22283-0

47. McMillan AG, May LE, Gaines GG, Isler C, Kuehn D. Effects of Aerobic Exercise during Pregnancy on 1-Month Infant Neuromotor Skills. *Med Sci Sports Exerc*. 2019;51(8):1671-1676. doi:10.1249/MSS.0000000000001958

48. Menichini D, Petrella E, Dipace V, Di Monte A, Neri I, Facchinetti F. The impact of an early lifestyle intervention on pregnancy outcomes in a cohort of insulin-

resistant overweight and obese women. *Nutrients*. 2020;12(5):1-8. doi:10.3390/nu12051496

49. Mortaji N, Krzeczkowski J, Atkinson S, Amani B, Schmidt LA, Van Lieshout RJ. Early neurodevelopment in the offspring of women enrolled in a randomized controlled trial assessing the effectiveness of a nutrition plus exercise intervention on the cognitive development of 12-month-olds. *J Dev Orig Health Dis*. 2023;14(4):532-539. doi:10.1017/S204017442300020X

50. Murtezani A, Paçarada M, Ibraimi Z, Nevzati A, Abazi N. The impact of exercise during pregnancy on neonatal outcomes: a randomized controlled trial. *J Sports Med Phys Fitness*. 2014;54(6):802-808.

51. Nascimento S, Surita F, Parpinelli M, Siani S, Pinto e Silva J. The effect of an antenatal physical exercise programme on maternal/perinatal outcomes and quality of life in overweight and obese pregnant women: a randomised clinical trial. *BJOG*. 2011;118(12):1455-1463. doi:10.1111/j.1471-0528.2011.03084.x

52. Oostdam N, van Poppel M, Wouters M, et al. No effect of the FitFor2 exercise programme on blood glucose, insulin sensitivity, and birthweight in pregnant women who were overweight and at risk for gestational diabetes: results of a randomised controlled trial. *BJOG*. 2012;119(9):1098-1107. doi:10.1111/j.1471-0528.2012.03366.x

53. Perales M, Calabria I, Lopez C, Franco E, Coteron J, Barakat R. Regular Exercise Throughout Pregnancy Is Associated With a Shorter First Stage of Labor. *Am J Health Promot*. 2016;30(3):149-154. doi:10.4278/ajhp.140221-QUAN-79

54. Price BB, Amini SB, Kappeler K. Exercise in pregnancy: effect on fitness and obstetric outcomes-a randomized trial. *Med Sci Sports Exerc*. 2012;44(12):2263-2269. doi:10.1249/MSS.0b013e318267ad67

55. Raper MJ, McDonald S, Johnston C, et al. The influence of exercise during pregnancy on racial/ethnic health disparities and birth outcomes. *BMC Pregnancy Childbirth*. 2021;21(1). doi:10.1186/s12884-021-03717-5

56. Rodriguez-Blanque R, Carlos Sanchez-Garcia J, Manuel Sanchez-Lopez A, et al. Influence of physical exercise during pregnancy on newborn weight: a randomized clinical trial. *Nutr Hosp*. 2017;34(4):834-840. doi:10.20960/nh.1095

57. Rodriguez-Blanque R, Carlos Sanchez-Garcia J, Manuel Sanchez-Lopez A, Exposito-Ruiz M, Jose Aguilar-Cordero M. Randomized Clinical Trial of an Aquatic Physical Exercise Program During Pregnancy. *JOGNN-JOURNAL OF OBSTETRIC GYNECOLOGIC AND NEONATAL NURSING*. 2019;48(3):321-331. doi:10.1016/j.jogn.2019.02.003

58. Roland CB, Knudsen S dP., Alomairah SA, et al. Effects of prenatal exercise on gestational weight gain, obstetric and neonatal outcomes: FitMum randomized controlled trial. *BMC Pregnancy Childbirth*. 2023;23(1). doi:10.1186/s12884-023-05507-7

59. Ruchat SM, Davenport MH, Giroux I, et al. Nutrition and Exercise Reduce Excessive Weight Gain in Normal-Weight Pregnant Women. *Med Sci Sports Exerc*. 2012;44(8):1419-1426. doi:10.1249/MSS.0b013e31825365f1

60. Ruiz JR, Perales M, Pelaez M, Lopez C, Lucia A, Barakat R. Supervised exercise-based intervention to prevent excessive gestational weight gain: a randomized controlled trial. *Mayo Clin Proc*. 2013;88(12):1388-1397. doi:10.1016/J.MAYOCP.2013.07.020

61. Seneviratne S, Jiang Y, Derraik J, et al. Effects of antenatal exercise in overweight and obese pregnant women on maternal and perinatal outcomes: a randomised controlled trial. *BJOG*. 2016;123(4):588-597. doi:10.1111/1471-0528.13738

62. Sklempe Kokic I, Ivanisevic M, Biolo G, Simunic B, Kokic T, Pisot R. Combination of a structured aerobic and resistance exercise improves glycaemic control in pregnant women diagnosed with gestational diabetes mellitus. A randomised controlled trial. *Women and Birth*. 2018;31(4):e232-e238. doi:10.1016/j.wombi.2017.10.004

63. Stafne SN, Salvesen KÅ, Romundstad PR, et al. Regular exercise during pregnancy to prevent gestational diabetes: A randomized controlled trial. *Obstet Gynecol*. 2012;119(1):29-36. doi:10.1097/AOG.0b013e3182393f86

64. Strom CJ, McDonald SM, Remchak MM, et al. Maternal Aerobic Exercise, but Not Blood Docosahexaenoic Acid and Eicosapentaenoic Acid Concentrations, during Pregnancy Influence Infant Body Composition. *Int J Environ Res Public Health*. 2022;19(14). doi:10.3390/ijerph19148293

65. Tomić V, Sporiš G, Tomić J, et al. The effect of maternal exercise during pregnancy on abnormal fetal growth. *Croat Med J*. 2013;54(4):362-368. doi:10.3325/cmj.2013.54.362

66. Wang C, Wei Y, Zhang X, et al. A randomized clinical trial of exercise during pregnancy to prevent gestational diabetes mellitus and improve pregnancy outcome in overweight and obese pregnant women. *Am J Obstet Gynecol*. 2017;216(4):340-351. doi:10.1016/j.ajog.2017.01.037

67. Yang H, Zhao Y, Tu J, Chang Y, Xiao C. Effects of Antenatal Lifestyle Interventions in Pregnant Women with Normal Body Mass Index. *Iran J Public Health*. 2023;52(2):381-388.

68. Yang X, Wang G, Liu N, et al. Mediating effect of gestational weight gain on the preventive effect of exercise during pregnancy on macrosomia: a randomized clinical trial. *BMC Pregnancy Childbirth*. 2024;24(1). doi:10.1186/s12884-024-06527-7

69. Yekefallah L, Namdar P, Dehghankar L, Golestaneh F, Taheri S, Mohammadkhaniha F. The effect of yoga on the delivery and neonatal outcomes in nulliparous pregnant women in Iran: a clinical trial study. *BMC Pregnancy Childbirth*. 2021;21(1). doi:10.1186/s12884-021-03794-6
